# Origins of Reactivity in SAM-Utilizing Ribozyme SAMURI-Catalyzed RNA Alkylation

**DOI:** 10.1021/jacs.6c08579

**Published:** 2026-07-15

**Authors:** Julie Puyo-Fourtine, Yanan Du, Erika McCarthy, Şölen Ekesan, Darrin M. York

**Affiliations:** Laboratory for Biomolecular Simulation Research, Institute for Quantitative Biomedicine and Department of Chemistry and Chemical Biology, 242612 Rutgers University, Piscataway, New Jersey 08854, United States

## Abstract

Unlocking the design principles of programmable RNA catalysts capable of site-specific chemical modification is critical for expanding the functional and therapeutic potential of RNA. The SAM analogue-utilizing ribozyme (SAMURI) enables site-specific RNA alkylation using either S-adenosylmethionine (SAM) or the synthetic cofactor propargylic Se-2,6-diaminopurinribosyl-selenomethionineamide (ProSeDMA), yet the molecular determinants of its reactivity remain incompletely understood. Here, we combined molecular dynamics, 3D-RISM solvation analysis, alchemical free energy calculations, quantum p*K*_*a*_ shift predictions, and *ab initio* QM/MM free energy simulations to characterize the conformational and electronic factors that govern catalysis. Simulations show that, although the global fold of SAMURI remains stable in solution, the formation of catalytically competent near-attack configurations is rare, indicating that the observed rate depends on access to a minor fraction of these reactive conformations (*f*_react_). A putative Mg^2+^ binding site between the SAM carboxylate and the G30 phosphate, together with a hydrogen bond between the cofactor α-amine and U8:O2, enriches *f*_react_. QM/MM simulations support an S_N_2-like alkyl transfer mechanism and show that ProSeDMA reacts more readily than SAM primarily due to its more favorable electronic leaving group properties that enhance the intrinsic rate (*k*_int_). Atomic substitutions at A52 that tune the N3 p*K*_*a*_ enhance nucleophilicity, further lower the activation barrier, and increase *k*_int_. Together, these results show that SAMURI catalysis is governed by a combination of conformational preorganization and electronic effects, providing a framework to guide the design of new programmable RNA alkyltransferases.

## Graphical abstract

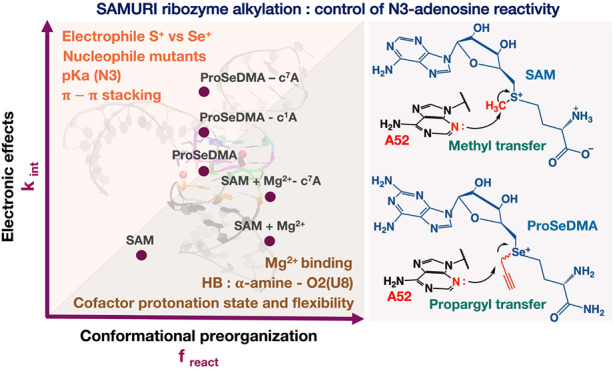

## Introduction

Nucleic acids can fold into complex three-dimensional architectures with catalytic activity, enabling them to function as enzymes in the absence of proteins—the so-called *ribozymes*.^1^ Their discovery overturned the long-standing view that only proteins could serve as biological catalysts^2–4^ and provided key support for the RNA world hypothesis, which posits that RNA was the primary self-replicating biomolecule in early life.^5,6^ Naturally occurring ribozymes are found across all kingdoms of life, where they participate in essential biological processes such as protein synthesis and tRNA maturation.^7,8^ Many also exhibit sequence-specific RNA cleavage activity that can be harnessed for therapeutic applications, including anticancer strategies targeting tumor-specific RNAs, as well as biosensing technologies.^9,10^

Beyond their biological functions, ribozymes provide a unique window into the chemical capabilities of RNA. However, natural ribozymes possess a limited catalytic repertoire, functioning primarily as nucleolytic catalysts that promote self-cleavage or ligation reactions and are largely restricted to phosphoryl-transfer chemistry, with only a few examples extending to peptide-bond formation.^11–14^

A broader range of reactions would have been required to sustain primitive metabolism, including C–C and C–N bond formation needed for the synthesis of molecular precursors, yet such activities are absent in known natural ribozymes.^6,15–17^ Artificial ribozymes that catalyze these reactions therefore provide a powerful platform for exploring plausible pathways of early metabolic evolution and the broader catalytic potential of RNA.

In the early 1990s, it was demonstrated that catalytic RNAs could be evolved toward new activities through iterative cycles of mutation, selection, and amplification.^18,19^ This *in vitro* approach, inspired by aptamer discovery, starts from large libraries of randomized RNA sequences challenged with a specific chemical task and enriches rare active sequences through successive rounds of selection.^20^ Over the past three decades, these methods have yielded ribozymes capable of RNA ligation and polymerization^21,22^ as well as catalyzing small molecule transformations, including Diels–Alder cycloadditions,^23^ aldol condensations,^24^ Michael additions^25^ and site-specific alkylations.^9,26–29^ These findings demonstrate that RNA catalysis extends far beyond what has been observed in nature so far.

Among these reactions, alkyl group transfer is of particular biological and technological interest. In living systems, alkylation of nucleic acids is a pervasive modification that can modulate base pairing, alter charge and hydrogen-bonding patterns, influence RNA folding, and regulate interactions with proteins.^30^ In tRNA and rRNA, specific methylations help stabilize structure and support accurate translation.^31,32^ More generally, alkyl transfer reactions enable the introduction of reactive handles for fluorescence labeling, affinity capture, or chemical modification.^33–35^

Such reactions are predominantly catalyzed by protein methyltransferases using S-adenosylmethionine (SAM) as the alkyl donor.^36^ However, these enzymes typically recognize conserved sequence motifs or structural folds, making site-specific RNA modification challenging.^27,37^ Several studies have demonstrated that RNA can be evolved to bind an alkyl donor and promote selective transfer, including the self-biotinylating ribozyme,^9^ the SAM-dependent guanine N7 methyltransferase SMRZ-1,^27^ the *O* ^6^-alkylguanine-dependent adenine N1 alkyltransferases MTR1 ^26,38–40^ and RACR,^28^ and the *O* ^6^-benzylguanine-dependent cytosine N4 alkyltransferase CSAR.^41^

Recently, the SAM analogue-utilizing ribozyme (SAMURI) was shown to form a fully RNA-based active site capable of site-specific alkylation.^33,42^ Evolved *in vitro* from an RNA library, this ribozyme uses a chemically stabilized and bioorthogonal analogue of SAM, propargylic Se-2,6-diaminopurinribosyl-selenomethionineamide (ProSeDMA), to transfer a propargyl group to the N3 position of a specific adenosine in a target RNA. The resulting alkyne provides a small reactive handle for click chemistry, allowing subsequent labeling with fluorophores, affinity tags, and related probes.^35^ SAMURI remains active at physiological Mg^2+^ concentrations and retains catalytic activity in mammalian cells, making direct intracellular RNA labeling possible. It can also use natural SAM to produce N3-methyladenosine (m^3^A), extending its reactivity beyond the engineered cofactor.

Structural studies^33,42^ showed that SAMURI adopts a compact three-helix junction in which the catalytic core is organized into four stacked layers that preorganize the reactive groups for chemistry. The upper layer contains the donor ProSeDMA in a base triple, where stacking interactions and hydrogen bonds orient the reactive selenium center for nucleophilic attack. In the next layer, the target adenosine A52 is positioned in a second base triple between P1 and P3, aligning its N3 atom with the cofactor in an S_N_2-like arrangement. Below this, two base pairs stabilize the fold and a lower layer further reinforces the stack. This architecture helps explain both the efficiency and the selectivity of SAMURI by juxtaposing the alkyl donor with the reactive nucleophile while disfavoring unproductive geometries (Figure 1). Two hydrated Mg^2+^ ions also contribute to the organization of the junction by linking backbone phosphates and supporting the kink that inserts A52 into the active site. More generally, prior structural and computational studies have shown that metal ions and their hydration shells play central roles in organizing RNA folds and shaping catalytically relevant conformations.^43–45^ Beyond their structural roles, divalent Mg^2+^ ions near the active site can also directly participate in the chemical steps of catalysis by reducing reaction barriers through electrostatic stabilization of the transition state, modulation of active site p*K*_*a*_ values, activation of nucleophiles, or direct involvement in general acid–base catalysis.^46–54^

**Figure 1:**
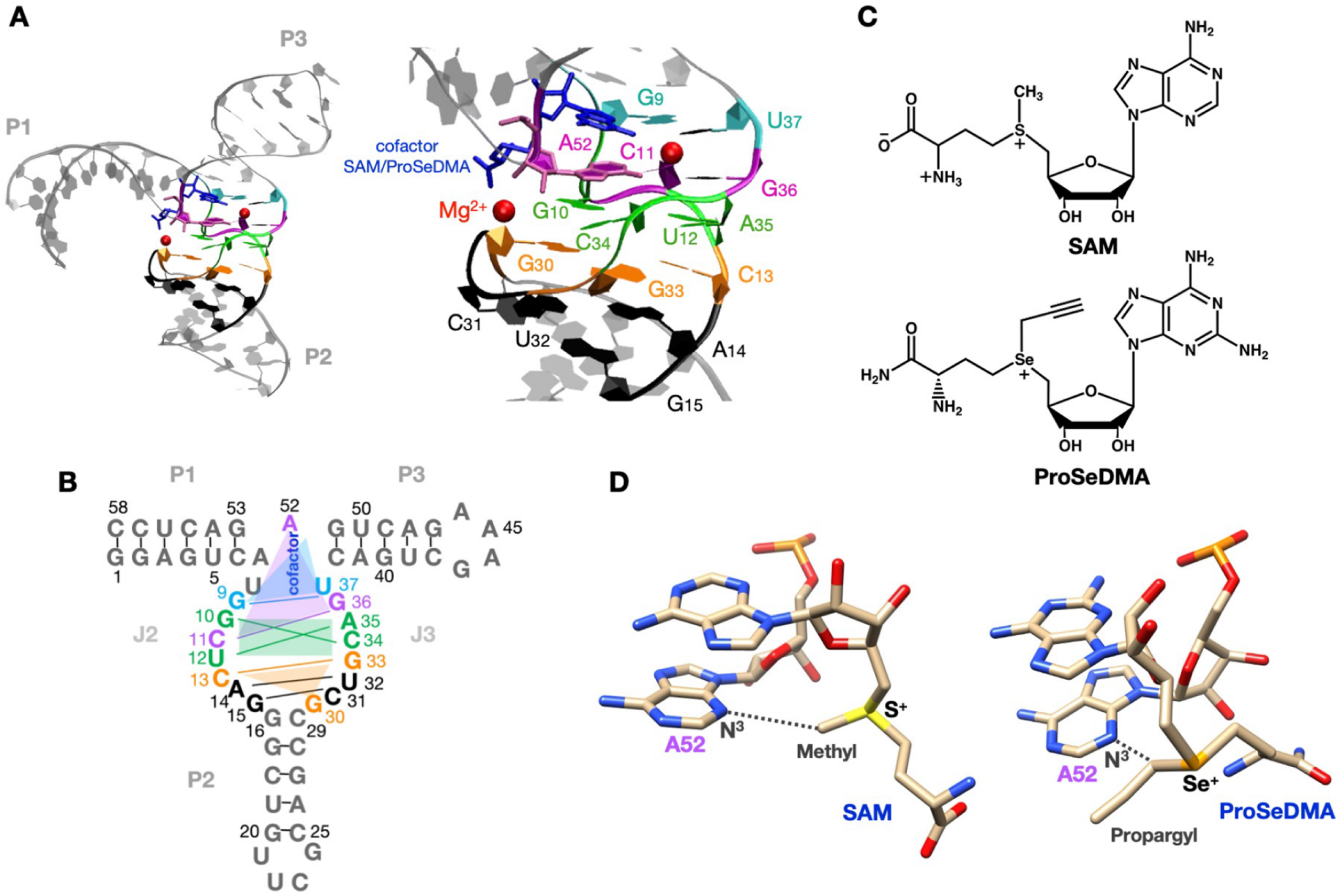
SAMURI ribozyme structure and cofactors. (A) Representation of SAMURI with the catalytic core highlighted by its four stacked layers: the *cofactor layer* (blue) defined by a base triple, the *reaction layer* (magenta) where the target adenosine (A52) engages in a base triple, the *stabilizing layer* (green) composed of two coplanar Watson–Crick base pairs, and the *bottom layer* (orange) formed by another base triple. The zipper region is shown in black, while helices P1, P2, P3, and loop regions are in gray. (B) Secondary structure of the 58-nt SAMURI construct, colored according to the scheme in (A). (C) Chemical representation of the two cofactors used in this study: S-adenosylmethionine (SAM), and the synthetic analog Propargylic Se-2,6-diaminopurin-ribosyl-selenomethionineamide (ProSeDMA). (D) Active site views with the two cofactors: SAM (left) enabling methyl transfer and ProSeDMA (right) enabling propargyl transfer.

Apart from its biochemical interest, SAMURI also provides a useful system for studying RNA-ligand interactions by computational approaches. This type of atomistic analysis is particularly valuable for RNA systems, where conformational heterogeneity, solvation, and ion binding are often tightly coupled determinants of structure and reactivity.^43,55^ Its relatively simple catalytic mechanism makes it well suited for examining cofactor conformational behavior in the active site, the determinants of cofactor stabilization, and the effects of mutations or cofactor modifications on reactivity.

In this work, we apply a multiscale^44,56–58^ computational enzymology framework^59,60^ to probe SAMURI’s catalytic mechanism at atomistic resolution. Molecular dynamics (MD) simulations were performed on the ribozyme-substrate-cofactor complex with either SAM or ProSeDMA, enabling a direct comparison of the two cofactors in the same structural context. *Ab initio* quantum mechanical/molecular mechanical (QM/MM) calculations were then used to explore the reaction coordinate, characterize the effect of mutations at the target adenosine A52 and evaluate the energy barriers. Alchemical free energy calculations were used to assess whether a divalent ion stabilizing the SAM cofactor is favorable. This combined approach corroborates the experimentally proposed S_N_2-like mechanism, reveals key factors governing N3 selectivity, and provides general principles for designing programmable RNA alkyltransferases for applications in chemical biology, RNA labeling, and synthetic biology.

## Methods

### Molecular Dynamics Simulations

#### System Preparation

Two main systems were prepared: the SAMURI ribozyme bound to either S-adenosylmethionine (SAM) or Propargylic Se-2,6-diaminopurin-ribosyl-selenomethionineamide (ProSeDMA). Starting structures were generated using VMD^61^ by superimposing the asymmetric units from the respective X-ray crystal structures (PDB codes 9FN2 for SAM and 9FN3 for ProSeDMA).^42^ Combining structural information from both subunits allowed localization of five Mg^2+^ ions in the SAM-bound structure and three in the ProSeDMA-bound structure. For each case, the simulated ribozyme was the subunit with the highest number of base pairs: the second subunit in 9FN2 and the first subunit in 9FN3. Two guanosine diphosphates originally present to improve crystal packing between subunits were removed. All crystallographically resolved water molecules within the Mg^2+^ hydration shells were retained. The SAMURI–cofactor complexes were parametrized with the ff99OL3 RNA force field^62,63^ and solvated in a truncated octahedron TIP4P-Ew water box.^64^ In all cases, ion counts ensured overall neutrality and reproduced a physiological 140 mM NaCl concentration, using monovalent ion parameters^65^ compatible with TIP4P-Ew and including 12–6–4 corrections.^66,67^ For Mg^2+^ ions, the corrections of Panteva et al. were applied to improve the description of RNA–Mg^2+^ interactions.^68,69^ The detailed composition of each simulated system is reported in Table S1.

#### Cofactors and Simulated Systems

Classical molecular dynamics simulations were carried out for four cofactor states (Figure 2A): decarboxylated S-adenosylmethionine (SAM dcAdoMet), SAM, SAM+Mg^2+^, and ProSeDMA. QM/MM simulations were performed for all systems except SAM dcAdoMet and were further extended to the SAM+Mg^2+^—c^7^A52, ProSeDMA—c^7^A52 and ProSeDMA—c^1^A52 variants, where c^7^A52 and c^1^A52 denote 7-deazaadenosine and 1-deazaadenosine at position 52, respectively (Figure 3). Full system compositions are provided in Table S1.

**Figure 2:**
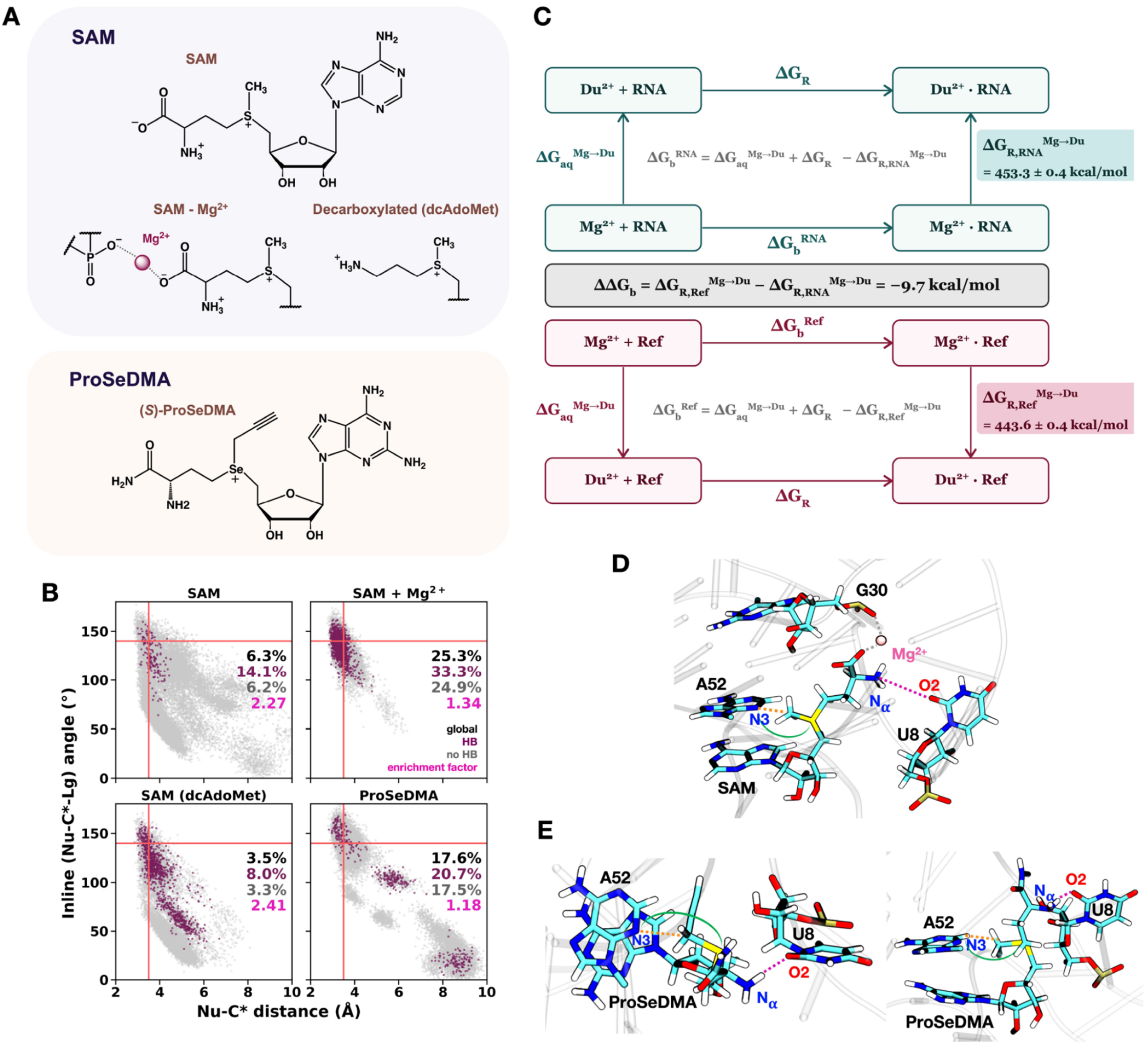
Active site organization facilitates alkyl transfer. (A) Chemical structures of S-adenosylmethionine (SAM), SAM coordinated to Mg^2+^ (SAM-Mg^2+^), decarboxylated S-adenosylmethionine (dcAdoMet), and propargylic Se-2,6-diaminopurin ribosyl-selenomethionineamide (ProSeDMA). (B) Distributions of inline attack angle (Nu-C*-Lg) and distance (Nu-C*), where the nucleophile (Nu) is A52:N3, C* is the electrophilic α-carbon of the cofactor, and the leaving group (Lg) is S (SAM, dcAdoMet) or Se (ProSeDMA). The fraction of reactive frames from four 250 ns replicas is shown in black (distance <3.5 Å, angle >140°); frames with or without the α-amino–O2­(U8) hydrogen bond are shown in burgundy and gray, respectively. (C) Thermodynamic cycle used to evaluate the relative binding free energy of the proposed Mg^2+^-binding site in SAMURI relative to a reference dinucleotide monophosphate, yielding ΔΔ*G*_b_. Here, Du^2+^ denotes a noninteracting dummy ion. $\Delta G_{aq}^{Mg\to Du}$ corresponds to the decoupling free energy of the ion in the bulk, $\Delta G_{R,\mathrm{RNA}}^{Mg\to Du}$ and $\Delta G_{R,\mathrm{Ref}}^{Mg\to Du}$ are the corresponding bound-state decoupling free energies, and ΔG_R_ is the analytical restraint contribution. (D) Active site with SAM coordinated to the proposed Mg^2+^. (E) Active site with ProSeDMA (top and side views). In D and E, the nucleophilic attack distance (orange), attack angle (green), and the α-NH_2_–O2­(U8) hydrogen bond (purple dashed lines) are indicated.

**Figure 3:**
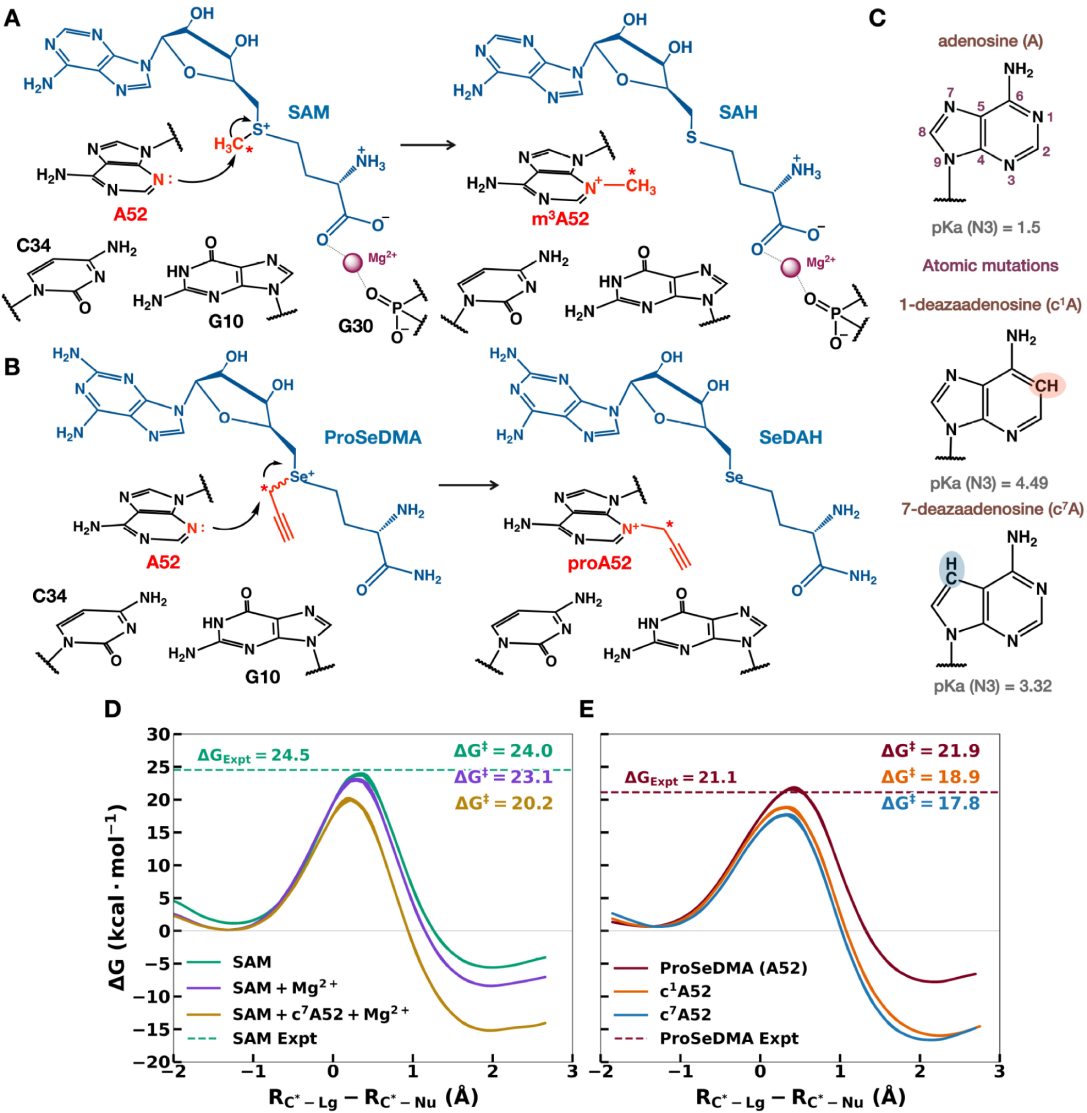
Alkyl-group transfer reactions and QM/MM free energy profiles. (A) ChemDraw representation of the methyl transfer from SAM to N3 of A52. (B) ChemDraw representation of the propargyl transfer from ProSeDMA to N3 of A52. (C) p*K_a_* (N3) values for adenosine (A) from ref 122, 1-deazaadenosine (c^1^A) and 7-deazaadenosine (c^7^A) estimated from shifts relative to A calculated (Tables S4, S5). (D, E) *ab initio* QM/MM free energy profiles projected onto the reaction coordinate *R*_C*‑Lg_ – *R*_C*‑Nu_, with activation free energies (Δ*G* ^‡^). Profiles were obtained from umbrella sampling (32 windows, 32 ps per window). (D): SAM, SAM+Mg^2+^, SAM+c^7^A+Mg^2+^. (E) Comparison for A (ProSeDMA), c^1^A and c^7^A substrates. In both cases, the experimental values are represented in dashed lines.

#### Parameterization of Nonstandard Residues

Three ligands and two modified nucleic acid residues were parametrized for use in the simulations. A common protocol was applied to all five species to ensure consistency: each molecule was first geometry-optimized with Gaussian 16 ^70^ at the MP2/6–31G* level,^71^ followed by electrostatic potential calculations at the HF/6–31G* level. Partial atomic charges were then determined using the RESP procedure.^72^ Atom types and force field parameters were assigned through Antechamber.^73^ SAM-like residues were parametrized using GAFF2 atom types. ProSeDMA-like residues were described using AMBER atom types, consistent with standard AMBER OL3 nucleic acid parameters,^62^ to improve the geometry of the diaminopurine base. Bonded and nonbonded parameters for selenium were refined from sulfur analogies and MP2/6–31G*^71^ calculations on model compounds (Table S2 and Figure S1) (see Supporting Information for more details).

#### MD Simulation Protocol

All classical molecular dynamics (MD) simulations were performed with AMBER24 ^74^ using GPU-accelerated MD.^75,76^ Temperature was controlled with a Langevin thermostat^77^ (γ = 5 ps^–1^) and pressure with Monte Carlo barostat^78^ (τ = 1 ps) at 300 K and 1 atm. Long-range electrostatics were treated with particle mesh Ewald^79,80^ using a 12 Å real-space cutoff and covalent bonds involving hydrogen were constrained with SHAKE.^81^ A 1 fs time step was used during equilibration. Systems were equilibrated through a multistep protocol including restrained minimization, solvent relaxation, heating, density equilibration, and progressive release of positional and mechanistic restraints consistent with previous works.^39,82^ Global positional restraints were initially applied to solute heavy atoms, together with additional mechanistically relevant restraints defined through the DISANG file. For systems containing ProSeDMA, extra restraints were introduced to preserve the active site stacking and hydrogen bond network during equilibration. Full restraint definitions and force constants are provided in the Supporting Information.

After equilibration, four independent 350 ns production trajectories were generated for each system in the NPT ensemble at 300 K and 1 atm. No global positional restraints were applied to the RNA or cofactor heavy atoms during production; however, harmonic restraints (*k* ≈ 40 kcal mol^–1^ Å^–2^) were maintained on the two crystallographic Mg^2+^ ions in the systems with SAM and upper-wall distance restraints (*k* = 30 kcal mol^–1^ Å^–2^) were retained for the G9–U37 and ProSeDMA–U37 interactions in the ProSeDMA system (G9­(O6)–U37­(O2′), G9­(N1)–U37­(O2), and ProSeDMA­(N6)–U37­(O4)); full details are provided in the Supporting Information (MD Simulation Protocol). The first 100 ns of each trajectory were discarded for the analysis. Hydrogen mass repartitioning^83^ (HMR) was employed during production, enabling a 4 fs time step with SHAKE.^81^ Representative equilibrated structures were then selected for subsequent QM/MM simulations based on an in-line fitness score. Further details are given in the Supporting Information.

### 3D-RISM

Single-point three-dimensional reference interaction site model (3D-RISM)^84,85^ calculations were performed on the selected asymmetric units stripped of solvent and ions including Mg^2+^. First, site–site solvent susceptibilities for a solution of 10 mM Mg^2+^, 140 mM Na^+^, and 160 mM Cl^–^ (32,768 grid points, 0.025 Å spacing) were determined from dielectrically consistent RISM (DRISM) with the rism1d program.^86^ The modified direct inversion of the iterative subspace (MDIIS) approach was used to iteratively solve the DRISM equation with PSE2 closure to a residual tolerance of 10^–12^ at 298 K and a dielectric constant of 78.497 for bulk water. A constant density approach was used, and the density of water was assumed to be 55.296 M using the SPC/E water model.^87^ Single-point 3D-RISM was performed on a 200 × 200 × 200 Å^3^ grid with a 100 × 100 × 100 Å^3^ solvation box without solvent–solvent interaction cutoff. PSE1 closure was used with a residual tolerance of 10^–4^.

### Alchemical Free Energy Simulations of Mg^2+^ Binding

Absolute binding free energy (ABFE) simulations for Mg^2+^ were computed using AMBER24 ^74,76^ and analyzed using FE-ToolKit^88^ distributed within the AmberTools.^75^ Two systems were considered (Figure 2C): (i) Mg^2+^ bound to the G30 phosphate in the SAM-bound ribozyme and (ii) a reference dinucleotide monophosphate system in which Mg^2+^ is bound to a phosphate group positioned between a guanine and a cytosine residue. The ribozyme system was extracted from the classical MD simulations described above, whereas the reference system was constructed separately at the same NaCl concentration of 140 mM.

The free energy simulations were performed on three trials (Figure S2) using the GPU-accelerated free energy engine in AMBER^89^ that integrates advanced features described in detail elsewhere.^90,91^ Simulations were performed using an optimized alchemical transformation pathway with second-order smoothstep softcore potential^92^ and 36-window optimized phase space overlap^93^ λ schedule and ACES^94,95^ enhanced sampling approach (gti_add_sc flag set to 25). Within the ACES approach, Hamiltonian replica exchange^96–100^ attempts were made every 20 steps. A λ-dependent harmonic restraint between Mg^2+^ and the phosphate oxygen was applied during the transformation, and its contribution in the dummy state^101^ removed analytically.^102^ Production sampling was performed after rigorous end-state equilibration for 5 ns in the NPT ensemble. Free energies were estimated with MBAR^103^ using a variational method^104^ implemented within FE-ToolKit package^88^ distributed with AmberTools.^75^ Full details about the equilibration procedures, λ schedules, and supporting analyses are provided in the Supporting Information.

### *Ab Initio* QM/MM Simulations

All QM/MM simulations were conducted using Amber.^105,106^ *Ab initio* QM/MM umbrella sampling simulations at the PBE0/6–31G* level^107^ were used to investigate methyl and propargyl transfer reactions with long-ranged electrostatic interactions treated rigorously with the ambient potential composite Ewald method.^108^ This level of theory has been employed in several recent studies of ribozyme systems^39,40,109,110^ and has been shown to provide a favorable balance between accuracy and computational efficiency for modeling phosphoryl and alkyl transfer reactions.

Starting configurations for *ab initio* QM/MM sampling were generated using two protocols depending on the cofactor. For SAM systems, windows were obtained sequentially using the third-order density-functional tight-binding (DFTB3) Hamiltonian and the 3OB-3–1 parameter set,^111–114^ employing semiempirical Ewald electrostatics,^115^ with 3 ps of equilibration per window at this level, followed by 1 ps of equilibration at the PBE0 level.^107^ For ProSeDMA systems, windows were generated directly at the PBE0 level (as DFTB3 parameters are not available for Se) using 0.5 ps of equilibration per window followed by 1 ps of final equilibration. In all cases, the reported free energy profiles are based on a cumulative total of 32 ps of *ab initio* QM/MM sampling per window at the PBE0/6–31G* level,^107,116^ with long-range electrostatic interactions treated using the ambient potential composite Ewald method.^108^

All QM/MM simulations used 10 Å real-space cutoffs and were carried out with a 1 fs time step without use of SHAKE in the QM region. The QM-MM boundary was treated with the hydrogen link atom approach.^117,118^

The reactive state is defined by SAM or ProSeDMA, with the positively charged sulfur or selenium center linked to a methyl or propargyl group, respectively, oriented toward the adenine 52. The product state corresponds to the adenine base methylated or propargylated at the N3 position. The reactions were described by a single reaction coordinate corresponding to alkyl transfer,

1 $$\xi =R_{C^{*}-\mathrm{Lg}}-R_{C^{*}-\mathrm{Nu}}$$

where *R*_C*‑Lg_ is the distance between the transferring carbon and the leaving group, and *R*_C*‑Nu_ is the distance between the transferring carbon and the nucleophile. Nu is m^3^A52 and proA52, Lg is SAH and SeDAH, respectively (Figure 3). Umbrella sampling was performed using 32 windows spanning ξ_SAM_ ∈ [−1.8, 2.4] Å and ξ_ProSeDMA_ ∈ [−1.6, 2.5] Å, with 32 ps of sampling collected for each window. Free energy profiles were analyzed using a variational approach^119^ implemented within the FE-ToolKit package^88^ distributed with AmberTools.^75^

To assess the sensitivity of the reaction profiles to the QM region size, three QM region definitions were benchmarked at the PM3 level: *large*, *medium*, and *small* (Figure S3). PM3 was used for this benchmark because AM1 and DFTB3 parameters for selenium were not available in the present setup, while test calculations with xTB showed problematic behavior. Umbrella sampling was performed using 32 windows spanning ξ_SAM_ ∈ [−1.8, 2.4] Å and ξ_ProSeDMA_ ∈ [−1.6, 2.5] Å, with 60 ps of sampling collected for each window. Relative to the *large* QM region, the computed activation free energies differed by +0.3 and −0.4 kcal mol^–1^ for the *medium* and *small* SAM models, respectively, and by +0.9 and −0.5 kcal mol^–1^ for the corresponding ProSeDMA models. Because these differences remained below 1 kcal mol^–1^, the *small* QM region was selected for the much more computationally intensive production *ab initio* QM/MM simulations.

For SAM, the quantum region includes the adenine base of A52, the methyl group, the sulfur atom, and the entire tail of the cofactor, retaining the CH_2_ group adjacent to the sulfur center corresponding to carbon C5′ (cut between C5′ and C4′), for a total of 37 atoms (Figure S4). In the SAM+Mg^2+^ conformation, the quantum part was expanded to comprise the Mg^2+^ ion with four coordinating water molecules, the phosphate group of G30 (including C3′, C5′, and bound hydrogens), and the SAM tail up to the ribose, giving a total of 60 atoms (Figure S4). Regarding the system with SAM as a cofactor interacting with Mg^2+^ and the c^7^A52 mutation, the QM region remains the same, except for a modification at base A52 that introduces one additional atom, resulting in a total of 61 atoms. For ProSeDMA, the quantum region includes the propargyl group, the selenium atom, the two CH_2_ groups directly bonded to selenium, the adenine base of A52, and the underlying guanine base G10 to capture π–π stacking with the propargyl triple bond, totaling 42 atoms (Figure S4). The definition of the QM part for the c^1^A and c^7^A variants was the same, with a change at base A52 leading to an additional atom (CH instead of N), with a total of 43 atoms. Uncertainties are reported in Table S3 as bootstrap standard errors obtained by combining within-replica bootstrap resampling (−nboot 50) and ensemble averaging across replicas.^120^ Detailed QM region definitions, including the number of QM atoms for each system and the boundary choices, are provided in the Supporting Information.

### *Ab Initio* DFT Predictions of Modified Nucleobase p*K_a_* s

A recent study made computational predictions of the p*K*_*a*_ values at a number of positions for a wide range of modified nucleobases,^121^ including at the N3 position of adenosine and the c^1^A and c^7^A modifications of specific interest to the present work. This study reported a predicted p*K*_*a*_ value of 0.8 at the N3 position of adenosine, which differed somewhat from the experimentally derived value of 1.5.^122^ Motivated by the original work,^121^ we set out to perform calculations that would provide a more precise estimate of the p*K*_*a*_ shift at the N3 position of adenosine due to c^1^A and c^7^A atomic mutants, following the same protocol in which the ribose moiety was replaced by a methyl group to reduce computational cost.

We performed p*K*_*a*_ calculations on 15 sites of modified nucleobases (Table S4) closely related to adenosine, using electronic structure and implicit solvation methods in Gaussian 16.^70^ Geometry optimization and frequency calculations were performed both at the M06–2X/aug-cc-pVTZ and the PBE0/6–31G* ^107,116^ levels of theory^123,124^ with the SMD implicit solvation model.^125^ The former (higher-level of theory) was used to make quantitative predictions used in this work, whereas the latter was used to gauge the accuracy of the level of theory used in the QM/MM simulations.

Tight convergence criteria and an ultrafine integration grid were employed throughout. The p*K*_*a*_ values were computed using a free energy approach based on the Gibbs free energy difference between protonated and deprotonated species in aqueous solution (eq 2), where the standard free energy of the proton in water (−270.297 kcal/mol) was used as in other work.^121^ Because only the relative p*K*_*a*_ values (Δp*K*_*a*_) are considered, the contribution of the standard Gibbs free energy of the proton in water cancels and thus does not influence the calculated p*K*_*a*_ shifts. The deprotonation Gibbs free energy for a species AH is given as

2 $$\Delta G\left(AH\right)=G\left(A^{-}\right)+G\left(H^{+}\right)-G\left(\mathrm{AH}\right)$$

and the corresponding calculated p*K*_*a*_ values were determined as^121^

3 $$pK_{a}^{\mathrm{calc}}=\frac{\Delta G}{2.303\cdot \mathrm{RT}}$$

where *R* is the gas constant and *T* is the temperature (298 K).

Two linear regression models based on calculated p*K*_*a*_ values obtained at the two levels of theory were fit to the known experimental values^122,126–128^ to compare and improve predictions of similar molecules with unknown p*K*_*a*_ values (Figures S5–S7 and Table S5). The resulting regression model based on the M06–2X/aug-cc-pVTZ results is given by

4 $$pK_{a}^{\mathrm{model}}=0.671\left(pK_{a}^{\mathrm{calc}}\right)+2.021$$

Additional details regarding the compounds used and validation tests are given in the Supporting Information.

## Results

### Conformational Dynamics in Solution Indicate That the Active Ribozyme Is a Rare State

The conformational ensemble of SAMURI in the bound state was characterized in solution using molecular simulation. Specifically, the cofactors explicitly considered were SAM, a decarboxylated SAM variant (dcAdoMet) and ProSeDMA shown in Figure 2A. The available crystal structures capture a postcatalytic state with the alkylated target adenosine and the cofactor products bound in the active site.^33,42^ To generate models of the prereaction complexes, the product nucleoside and modified cofactor were replaced with their reactant counterparts and then relaxed and equilibrated (see Methods). Independent molecular dynamics simulations were then performed for each bound cofactor to generate conformational ensembles in solution (1 μs aggregate simulation time across four independent replicas of 250 ns each).

All simulations showed stable ribozyme dynamics after ∼100 ns of relaxation from the crystal environment, preserving the overall fold and catalytic pocket (Figures S8–S11). The solvated ribozyme exhibited greater fluctuations than those estimated from crystallographic B factors, especially at peripheral, solvent-exposed helix termini and loop regions that were stabilized by packing contacts in the crystal (Figure S12). The catalytic core, on the other hand, remained comparatively more structured and less dynamic with root-mean-square deviation (RMSD) generally less than 2.0 Å.

In order for the S_N_2 alkylation reaction to occur, the nucleophile (A52:N3) needs to be in close proximity to the electrophilic α carbon of the cofactor (designated C*) and form a nearly inline attack with the α carbon and leaving group (S in SAM, Se in ProSeDMA). Despite the structural stability of the active site, the relative positions of the reactive groups of the RNA and cofactor exhibit variation. Figure 2B shows the inline fitness distribution as a function of the inline attack angle and distance of the conformational ensembles in solution. The formation of a catalytically active state, characterized by a donor–acceptor distance of less than 3.5 Å and an inline attack angle greater than 140°, only occurs 6.3% of the time for SAM and 17.6% of the time for ProSeDMA. This implies that the formation of catalytically active conformations for these cofactors is fairly rare (Figures S13–S16). The correlation between observed reaction rates and “in-line fitness” was first discussed by Soukup and Breaker^129^ in the context of RNA stability. Here, we adopt distance and angle cutoffs consistent with previous studies of RNA enzymes^39,60,82,130^ to evaluate trends in reactivity arising from in-line fitness. A sensitivity analysis of the geometric definition of the reactive ensemble based on the long-time scale MD simulations is provided in Figures S17–S19 and Table S6. As discussed below, system-specific distance and angle cutoffs used to estimate the free energy contribution to the activation barrier (Table S7) and predicted rates are further refined from the corresponding distributions observed in the shorter QM/MM simulations (Figures S20, S21). A comparison to a harmonic model is also provided (Figure S22).

### A Predicted Mg^2+^ Ion Anchors the Tail and Enhances Inline Fitness

Initial simulations of SAMURI were carried out without introducing additional divalent ions beyond those resolved in the crystallographic structure. Under these conditions for the SAM cofactor, Na^+^ ions were observed in the vicinity of the carboxylate terminus of the tail and a nearby phosphate (G30). Ribozyme active sites are often electrostatically strained^86^ and recruit a threshold occupancy of cationic charge from solvent.^131^ We thus employed 3D-RISM^84,132^ molecular solvation theory, which has been demonstrated to be successful in predicting functional metal ion binding sites in RNA,^86,109,133^ to explore a possible Mg^2+^ binding mode. Analysis revealed a pronounced Mg^2+^ density maximum between the SAM carboxylate and the pro-*S*_P_ oxygen of G30, supporting this region as a plausible binding site (Figure 2D and Figure S23). Another pronounced Mg^2+^ density maximum has been identified between C11 and U12 (Figure S24).

We next sought to gain more quantitative computational evidence for the feasibility of the Mg^2+^ binding site suggested by 3D-RISM using alchemical free energy simulations^90,91^ with enhanced sampling^93–95^ (see Computational Methods in Supporting Information for details). The Mg^2+^ ion was initially positioned at the local maximum of the 3D-RISM density (Figure S23) and in direct coordination with both the carboxylate of the SAM cofactor and the pro-*S*_P_ oxygen of G30. Mg^2+^ ions are expected to bind fairly weakly to a single phosphate in, for example, a canonical RNA helix environment,^134,135^ whereas substantially stronger binding can emerge at specific RNA sites depending on the local structural and electrostatic context.^135,136^ The relative binding free energy, ΔΔ*G*_b_, of the Mg^2+^ ion in the proposed site in SAMURI with respect to an isolated dinucleotide monophosphate in solution was calculated to be −9.7 kcal/mol using the thermodynamic cycle in Figure 2C (Figure S2). This indicates that the proposed Mg^2+^ binding site in SAMURI is predicted to be considerably more favorable than weak binding to the dinucleotide monophosphate reference. Taken together, this provides support that a Mg^2+^ ion could be partially occupied at this site for the SAM cofactor.

A notable outcome of the simulations is the effect of Mg^2+^ binding at this site, which has broader implications for ribozyme reactivity. The introduction of Mg^2+^ results in clear stabilization of both the global fold and the active site, with reduced local fluctuations and overall more stable behavior (Figures S8–S9, S12–S14). Remarkably, the overall inline fitness increased 4-fold, from 6.3% in the absence of Mg^2+^ to 25.3% with Mg^2+^ bound (Figure 2B,D). In the absence of Mg^2+^, this increased flexibility manifests as a broadening of the reactive geometry distributions rather than stabilization of a distinct alternative conformation. The Nu–C* distance shifts toward larger values (5–6 Å) and becomes more diffuse, while the inline attack angle distribution broadens accordingly (Figure S13). In contrast, Mg^2+^ binding sharpens both distributions and enriches populations with inline angles >140° (Figure S14), consistent with the observed 4-fold increase in inline fitness. This improvement arises primarily from reduced fluctuations in the cofactor tail, which are further enhanced by hydrogen bonding between the protonated α-amine of the SAM cofactor and the O2 atom of U8. The broader implications of this interaction are discussed in the following subsection.

### An α-Amine···U8–O2 Hydrogen Bond Promotes Catalytically Competent Conformations

Building on the experimental study,^42^ a key contact was identified between the cofactor α-amine and the O2 atom of U8, which is apparent in the crystal structure. In that work, deletion of A7, leaving U8 as the sole linker nucleotide, was sufficient to support folding and catalytic activity; subsequent substitution of U8 by cytidine was similarly well tolerated, whereas purine substitutions reduced activity, giving the trend U ≈ C > A > G. These observations point to a preference for pyrimidines and suggest that the O2 atom of U8 (present in both U and C) may contribute to catalysis as a hydrogen bond acceptor, without being strictly required. Consistent with this interpretation, ProSeDAB and ProSeDMA, which retain the α-amine, display comparable experimental activity, whereas ProSeDMA–OH and ProSeDBA are substantially less active^42^ (see Figure S25).

To explore the role of U8:O2 in promoting inline fitness, simulations were performed for each of the cofactors shown in Figure 2A. Trajectories were clustered into sets according to the presence or absence of a hydrogen bond between the α-amine of the cofactors and U8:O2. Separate analysis of the inline fitness for both data sets enables the calculation of an enrichment factor, defined as the ratio of inline fitness values in the presence and absence of the hydrogen bond with U8:O2. In each case, the enrichment factors were all greater than unity, indicating that the presence of this hydrogen bond is correlated with enhanced inline fitness (Figure 2B). This effect is most pronounced in the least preorganized systems (SAM and dcAdoMet, enrichment factors 2.3–2.4), and more modest for the structured systems (SAM+Mg^2+^ and ProSeDMA, enrichment factors 1.2–1.3) that had greater overall inline fitness. In all cases explored in the current work, the origin of enhanced inline fitness is correlated with the introduction of order in the cofactor tail, and thus depends on the broader hydrogen bond interaction network of the cofactor with key residues in the active site (Table S8), of which U8:O2 plays a prominent role (Figures S13–S16).

Beyond the U8:O2 contact, the cofactor’s α-amine (Table S8) engages a broader network of interactions whose composition differs sharply with and without Mg^2+^. In the absence of Mg^2+^, this α-amine forms only transient contacts with several backbone oxygens, with no dominant partner (G53:O5′, 0.24; G53:OP2, 0.13; G9:OP1, 0.13; Table S8), consistent with a disordered tail. Upon Mg^2+^ binding, the network reorganizes around a dominant contact with G10:OP1 (occupancy 0.69), together with a secondary contact to A7:N7 (0.31), consistent with Mg^2+^ bridging the SAM carboxylate to the G30 phosphate region and rigidifying the tail. In ProSeDMA, whose terminal amide is neutral, the α-amine shows comparably diffuse contacts (G53:O4′, 0.28; G53:O5′, 0.21; G10:N7, 0.16; Table S8), again without Mg^2+^ coordination at this site. The additional preorganization observed for ProSeDMA instead arises from two distinct interactions elsewhere on the cofactor: the exocyclic N2-amine of the diaminopurine base, absent in adenine, which forms a hydrogen bond to O5′ of A52, and π–π stacking between the propargyl group and G10, which locally anchors the reactive Se–C* center.

### Conformational Preorganization and Electrostatic Tuning Shape the Free Energy Barrier for Alkyl Transfer

The observed rate constant for a pseudo-first-order enzymatic reaction can be modeled as *k*_obs_ = *f*_react_ · *k*_int_, where *f*_react_ is the fraction of the dynamical ensemble of the enzyme in a catalytically competent reactive conformation (henceforth referred to as the ″active state″), and *k*_int_ is the intrinsic rate departing from the enzyme in its active state. In general, the active state fraction is a function of reaction conditions^137^ (e.g., pH, ion environment, and temperature), whereas the intrinsic rate (sometimes referred to as the cleavage rate for RNA enzymes^138^ derives from the chemical steps of the reaction and depends only on temperature under the assumption that the mechanism itself is invariant to the other state variables. For a given enzyme, both *f*_react_ and *k*_int_ are expected to vary with different substrates. Assuming simple transition state theory (unit transmission coefficient,^139^ the intrinsic rate can be modeled as $k_{int}=\left(k_{B}T/h\right)e^{-\Delta G^{‡}/\left(k_{B}T\right)}$ , where Δ*G* ^‡^ is the activation free energy, *k*_B_ and *h* are the Boltzmann and Planck constants, respectively, and *T* is the Kelvin temperature. In the case of acid–base catalysis, experimental activity-pH profiles can often be used to extract kinetic parameters that can be used to estimate *f*_react_ and *k*_int_, although these may not always be uniquely determined due to the principle of kinetic ambiguity.^137^ The SAMURI does not employ acid–base catalysis, and only the experimentally observed rate constant, *k*_obs_, is available.^33,42^ Nonetheless, we can make an estimate of the free energy contribution of *f*_react_, designated Δ*G*_react_, to the overall activation barrier for SAM and ProSeDMA as follows.

In principle, if the QM/MM simulations could sufficiently sample the full in-line fitness landscape, the resulting free energy profiles would naturally include the Δ*G*_react_ contribution to the overall activation barrier, Δ*G* ^‡^. However, whereas the classical simulations accumulated ∼1 μs of sampling, the QM/MM simulations were limited to only 32 ps per window and therefore sampled only local fluctuations around the initial reactive conformation. To bridge these disparate time scales, we analyzed the in-line fitness distributions from the QM/MM trajectories to determine the range of geometries effectively sampled and thus incorporated into the QM/MM free energy barrier (Figures S20–S21). These ranges were then mapped onto the full MM distributions (Figures S17–S19) to estimate the fraction of the active-state ensemble represented in the QM/MM simulations for each cofactor. The corresponding free energy contribution was estimated as Δ*G*_react_ = −*RT* ln­(*f*_react_). As described in detail in the Supporting Information, this procedure resulted in Δ*G*_react_ values of 1.1, 0.6, and 0.1 kcal/mol for the SAM, SAM+Mg^2+^, and ProSeDMA systems.

The alkyl-transfer reactions considered here for SAM and ProSeDMA are shown schematically in Figure 3A,B. In the SAM system, the reaction corresponds to the transfer of a methyl group from sulfur to the N3 of A52, whereas in the ProSeDMA system, a propargyl group is transferred from selenium to the same nucleophilic center. In both cases, the reaction proceeds through an S_N_2 mechanism, with cleavage of the S/Se–C* bond and formation of the C*–N3 bond. The reaction progress was described by a single distance difference coordinate, *R*_C*‑Lg_ – *R*_C*‑Nu_, where C* is the reacting α carbon, Lg is the leaving group (S in SAM and Se in ProSeDMA), and Nu is the nucleophile (N3 of A52).

Free energy profiles^119^ from *ab initio* QM/MM simulations with full long-ranged Ewald electrostatics^108^ are shown in Figure 3D,E. The activation free energy barriers estimated from simple transition state theory using the observed experimental rate constants (24.5 and 21.1 kcal/mol for SAM and ProSeDMA, respectively^33,42^ are shown as horizontal dashed lines for reference. The QM/MM free energy profiles are shifted by the Δ*G*_react_ values described above such that they are factored into the profiles and overall Δ*G* ^‡^ values consistently (Table S7).

The calculated activation free energy for SAM is Δ*G* ^‡^ = 24.0 kcal/mol in the absence of Mg^2+^, and slightly lower, Δ*G* ^‡^ = 23.1 kcal/mol in the presence of Mg^2+^ (Figure 3D). The former value is in very close agreement with the experimental reference value of 24.5 kcal/mol.

The lowering of the activation free energy in the presence of the bound Mg^2+^ results from preferential electrostatic interactions with the ion in the product state relative to the reactant state. Specifically, in going from the reactant state to the product, a formal +1 charge migrates from the leaving group to the nucleophile position, which is located farther away from the Mg^2+^. This results in a modest lowering of the reaction free energy relative to the reaction in the absence of the Mg^2+^, and in accordance with expected linear free energy relations,^140–143^ also leads to an earlier transition state and a slight lowering of the forward activation free energy barrier.

The activation free energy for ProSeDMA is 21.9 kcal/mol, in close agreement with the reference value of 21.1 kcal/mol. Experimentally, the activation barrier for ProSeDMA is 3.4 kcal/mol lower than that of SAM, and the calculations reproduce this trend, predicting a reduction of 2.1 kcal/mol. This lower barrier for ProSeDMA is consistent with selenium’s larger size and greater polarizability relative to sulfur, which improve its leaving-group ability and facilitate Se–C bond cleavage.

### QM/MM Simulations Aid in the Interpretation of Atomic Mutagenesis Experiments and Enable New Predictions

Fundamental experimental studies of nucleic acid enzymes have provided a wealth of insight into their structural organization and catalytic strategies.^12,144–146,146^ In the original SAMURI study,^33^ variants at the A52 position were examined with the ProSeDMA cofactor, including 1-deazaadenosine (c^1^A) and 7-deazaadenosine (c^7^A). Both mutants supported ProSeDMA-dependent alkylation with apparent activities similar to the native A52 substrate, although quantitative rate constants were not reported. These results establish that the N1 and N7 positions of A52 are not strictly required for catalysis, but do not resolve whether either substitution enhances reactivity. Because each deaza substitution replaces an electron-withdrawing endocyclic nitrogen with a more electron-donating CH group, the electronic structure of A52—particularly the N3 nucleophile—is expected to be perturbed. To assess these effects, we estimated the N3 p*K*_*a*_ values of the deaza variants and computed QM/MM free energy profiles to predict their impact on activation barriers and intrinsic rates. The barrier reductions discussed below therefore provide a computational rationale for the experimental tolerance of c^1^A and c^7^A, while suggesting that electronic tuning at A52 may enhance SAMURI reactivity.

The activation free energy of c^1^A is 18.9 kcal/mol, whereas c^7^A is 17.8 kcal/mol (Figure 3E). Hence, these atomic mutants have barriers lower than the unmodified SAMURI by 3.0 and 4.1 kcal/mol, respectively. These trends are consistent with the expected correlation with the calculated microscopic p*K*_*a*_ values at the N3 position shown in Figure 3C.^121,122^

Indeed, both c^1^A and c^7^A analogs have upshifted N3 p*K*_*a*_ values (by 2.4–4.1 units) relative to the unmodified adenosine (Figure 3C). The resulting electron donation into the aromatic ring leads to increased nucleophilicity at the N3 position and preferentially stabilizes the product state making the reaction more exergonic. In accordance with linear free energy relationships,^140–143^ this lowers the forward barrier and shifts the transition state toward reactants (producing an ″earlier″ transition state). In addition to these electronic effects, there are some differences observed in the hydrogen bond network between the c^1^A and c^7^A simulations. Specifically, for c^1^A, the N7 position receives a hydrogen bond from the HO2′ of C34, whereas in the c^7^A simulation, this interaction is not possible. Rather, in the c^7^A simulation, the N6 exocyclic amine donates a hydrogen bond to the O6 of G36 which forms a network with the N4 of C11 and N1 of A52.

Given the results for the c^1^A and c^7^A atomic mutants for ProSeDMA, we set out to predict a modification in the SAM cofactor that would be practical and enhance ribozyme reactivity. In the SAMURI study,^33^ it was reported that the electrospray ionization (ESI)-MS trace of the alkylated c^1^A sample contained a depurinated fragment not seen in the c^7^A sample. This behavior can be rationalized by the classical depurination mechanism, in which protonation at N7 generates a cationic purine that weakens the *N*-glycosidic bond and promotes cleavage (Figure S26). By contrast, c^7^A prevents protonation at N7 and consequently blocks access to the depurination pathway.^33^ To avoid the unwanted side reaction with c^1^A, we focus on the c^7^A atomic mutant in the presence of a bound Mg^2+^ ion to explore the degree to which electronic effects might further enhance the reaction rate with the SAM cofactor. Since this mutation is expected to increase the p*K*_*a*_ at N3, it was of interest to test whether the geometric effects induced by Mg^2+^ and the electronic effects associated with the c^7^A substitution could have a multiplicative effect on the rate. Analogously to the ProSeDMA cofactor, the calculated free energy profile for the c^7^A atomic mutant for the SAM cofactor leads to stabilization of the product state, lowering of the activation barrier by 3.8 and 2.9 kcal/mol with respect to the unmodified cofactor in the absence and presence of bound Mg^2+^, respectively. These results suggest that atomic mutants of A52 that increase nucleophilicity at N3, together with structural elements that anchor the cofactor tail, may be fruitful in enhancing SAMURI reactivity.

## Discussion

The SAMURI is an *in vitro*-selected ribozyme that binds SAM and ProSeDMA cofactors to catalyze site-specific RNA alkylation, including the transfer of a propargyl group used as a tag for azide–alkyne click chemistry in RNA labeling.^33,42^ The SAMURI does not appear to engage in sophisticated acid or base chemistry to achieve catalysis. Rather, the SAMURI structure binds the cofactor and positions the α carbon (C*) in sufficiently close proximity to the alkylation site (N3 of A52) so that reactive conformations can be sampled. Simulations suggest that these reactive conformations, collectively referred to as the “active state″, are fairly rare, and the introduction of structural elements that stabilize these conformations would enhance the reactivity. In particular, results suggest that the low fraction of reactive frames observed for SAM is consistent with a more disordered cofactor tail in comparison with the ProSeDMA that appears somewhat better adapted to the active state ensemble. Because a minimal set of local restraints was retained to preserve experimentally observed active site interactions, the comparison between SAM and ProSeDMA should be interpreted with this limitation in mind, although these restraints do not act directly on the Nu–C* distance or Nu–C*–Lg angle used to define the active-state population.

This scenario is similar to that of the self-alkylating ribozyme^147,148^ (SAR), which catalyzes the alkylation of the N7 atom of a guanine by loosely binding a polyether ligand with a reactive terminal epoxide and positioning it to undergo nucleophilic attack and ring opening. Alternatively, this mechanistic strategy contrasts with that reported for the methyltransferase ribozyme^38,149^ (MTR1), in which the reactive arrangement is much more strongly biased toward an inline geometry.^39,40^ The MTR1 active site is substantially more preorganized for chemistry than in SAMURI, using a protonated cytosine residue as an acid catalyst and an elaborate hydrogen-bond network to position the reactive elements and stabilize the nucleobase leaving group. At a broader level, the principles emerging from this work, namely conformational preorganization, electrostatic organization assisted by metal ions and enrichment of reactive states, find conceptual resonance in RNA catalysts that rely on a protein partner. In type III CRISPR effector complexes, for instance, aspartate residues contributed by the protein coordinate catalytic Mg^2+^ ions, and the scaffold formed by the protein and the RNA together imposes flipping of the target base and an in-line attack geometry reminiscent of the hammerhead, pistol, and Varkud ribozymes.^150^ This may be viewed as a complementary strategy to the one described here: in that system, protein subunits help organize the RNA substrate’s own 2′-OH chemistry, whereas SAMURI relies on RNA tertiary structure and local electrostatics alone to organize a reaction that is not native to RNA.

Hence, building upon the MTR1 example, strategies to improve catalytic efficiency in SAMURI would involve introducing interactions that further stabilize the active state, or introducing environmental or chemical modifications to enhance the reactivity. Simulations predict that one such example of the former might be achieved by the binding of a Mg^2+^ ion interacting with the SAM carboxylate, which is consistent with structural motifs observed in other SAM-binding RNAs.^27,151–153^ As summarized in Figure S27, divalent ions in several RNA systems contribute directly to the organization of the cofactor tail by bridging the carboxylate to the RNA scaffold or to nearby nucleobases. For example, in the SAM-V riboswitch, Mg^2+^ connects the SAM carboxylate to a backbone phosphate,^151^ whereas in the SAM-I riboswitch, a divalent ion directly coordinates the cofactor carboxylate while also interacting with a neighboring guanine.^152^ A related principle is also observed in the SAM-dependent methyltransferase ribozyme, where a divalent metal coordinates the SAM-like tail together with neighboring nucleobase heteroatoms.^27^ Taken together, these examples support the idea that divalent ions can locally stabilize charge and reinforce the architecture of SAM-binding pockets through a range of coordination modes.

The 3D-RISM and alchemical free energy results support a plausible Mg^2+^ binding site bridging the SAM carboxylate and the G30 phosphate. We note that Mg^2+^ binding to an isolated dinucleotide monophosphate in solution is intrinsically weak; thus, the calculated ΔΔ*G*_b_ of −9.7 kcal mol^–1^ reflects binding relative to this reference rather than a strong absolute affinity. A relative thermodynamic cycle was used specifically to avoid the larger uncertainties associated with absolute divalent-ion binding free energies. We further note that nonpolarizable force fields can overestimate divalent-ion affinities due to neglected electronic polarization,^154,155^ and that crystal and solution environments differ substantially in ionic strength, packing contacts, and solvent dynamics, all of which may influence Mg^2+^ occupancy. Consistent with this, the site is not resolved in the crystal structure. Such partially occupied or dynamically exchanging Mg^2+^ ions are often crystallographically invisible, as illustrated by the Varkud satellite ribozyme, where a functionally important ion absent in the crystal was predicted computationally and later validated experimentally.^109^ Unlike that case, however, where Mg^2+^ strongly influenced general-base positioning and p*K*_*a*_, the effect here on the activation barrier is modest, suggesting a limited direct catalytic role. Nevertheless, the simulations support the idea that engineering a stronger Mg^2+^ site capable of more tightly anchoring the cofactor tail could enhance SAM-dependent catalysis. Finally, the modeled inner-sphere coordination to both the SAM carboxylate and G30 phosphate represents only one plausible binding mode; the site should therefore be viewed more generally as a region of favorable Mg^2+^ affinity, where both inner- and outer-sphere coordination remain possible.

The simulation results also implicate the importance of the α-amine···U8–O2 hydrogen bond in stabilizing the active state. This interaction appears to function primarily as a local organizing contact within the SAMURI active site and correlates with the enrichment of reactive conformations, consistent with a role of biasing the conformational ensemble toward catalytically competent states. This effect is most evident in the least preorganized SAM systems, whereas its relative contribution is attenuated in SAM+Mg^2+^ and ProSeDMA, where the cofactor is more conformationally constrained. These results support a model in which the α-amine···U8–O2 interaction contributes to catalytic organization as one of several cooperative local interactions.^42^

This functional importance of the α-amine of the cofactor finds a parallel in the recent structural and computational study of the 3-ACP transferase NAT in nocardicin biosynthesis.^156^ In that enzyme, which also uses SAM as alkyl donor, QM/MM calculations revealed that the Cα-amino group of SAM acts as a Brønsted base, assisted by E143, to facilitate S_N_2 attack at the Cγ of SAM, constituting the first reported case in which the amino group of SAM participates directly in catalysis. These findings highlight the α-amine of SAM as a functionally important moiety in alkyl transfer reactions, whether through direct chemical participation or conformational organization of the active state.

The free energy profiles reveal trends in the intrinsic reactivity of the chemical steps that can be rationalized through consideration of electronic effects. The activation barriers are lower for the ProSeDMA cofactor than for SAM owing to the electronic properties of Se that enable it to be an enhanced leaving group relative to S.^33,42^ In addition, the propargyl group of ProSeDMA is electron-withdrawing, which can better stabilize the partial positive charge on the electrophilic C* compared to SAM. This electron-withdrawing property is analogous to the enhanced reactivity of MTR1 in the presence of *O* ^6^-alkylguanine moieties carrying aromatic substituents on C*. Atomic mutants that increase electron donation into the nucleobase aromatic ring raise the microscopic p*K*_*a*_ and enhance the nucleophilicity of the N3 position, leading to lower barriers and a faster intrinsic rate.

This also suggests a possible design of a double c^1^Ac^7^A modification. Indeed, an intrinsic electronic p*K*_*a*_ estimate at the M06–2X/aug-cc-pVTZ level gives an N3 p*K*_*a*_ of 5.92 (Table S5) for c^1^Ac^7^A, higher than those calculated for c1A and c7A individually, suggesting that the combined modification could further enhance the nucleophilic character of N3. However, the single mutant simulations show that c^1^A and c^7^A are accommodated differently within the local hydrogen bond network around A52. In c1A, N7 accepts a hydrogen bond from the HO2′ group of C34, whereas this interaction is absent in c^7^A; in c^7^A, the N6 exocyclic amine instead donates a hydrogen bond to O6 of G36, coupled to a local network involving N4 of C11 and N1 of A52. Thus, although a c^1^Ac^7^A variant may provide additional electronic activation, its catalytic effect would depend on whether the active site interaction network can tolerate the simultaneous loss of the N1 and N7 hydrogen bond positions while maintaining reactive preorganization.

Alternatively, there may be ways of altering the active site electrostatic environment that lead to p*K*_*a*_ tuning at the A52:N3 position to enhance its reactivity. For example, the twister ribozyme^157–159^ is a small self-cleaving RNA that uses the N3 position of a conserved active site adenine (A1) to act as a general acid catalyst.^60,130,160^ However, the intrinsic p*K*_*a*_ of the N3 position of adenine is quite low (estimated to be 1.5,^122^ and hence, if unshifted, it would not be available in its protonated form required to act as an acid catalyst at near-neutral pH. The twister ribozyme active site has positioned two phosphates to dually coordinate the N6 exocyclic amine of A1, anchoring it in a position to act as a general acid by transferring a proton to the O5′ leaving group of the scissile phosphate. At the same time, these two anionic residues serve to shift the p*K*_*a*_ of A1 at the N3 position by approximately 3.5 units.^60,130^ It is possible that engineering similar interactions with the N6 exocyclic amine of A52 in SAMURI could further shift the p*K*_*a*_ and enhance the nucleophilicity at the N3 position.

## Conclusion

By integrating molecular dynamics, 3D-RISM solvation analysis, alchemical free energy calculations, quantum p*K*_*a*_ predictions, and *ab initio* QM/MM free energy simulations, we delineate the conformational and electronic determinants of SAMURI catalysis. The introduction of order into the cofactor tail through metal ion binding and/or hydrogen bond networks enriches the fraction of active-state conformations, and isofunctional chemical modifications at A52 that enhance nucleophilicity increase the intrinsic rate. Together, these results establish that the origin of catalysis derives from a synergy between conformational preorganization and electronic tuning, in which rare reactive conformations and intrinsic chemical reactivity jointly determine activity. This mechanistic framework provides general design principles for engineering programmable RNA catalysts for site-specific chemical modification, advancing their application in chemical biology and therapeutics.

## Supplementary Material



## Data Availability

Raw data, parameters, coordinates, input files, scripts, and trajectories related to the simulations performed in this study are available at https://zenodo.org/records/20027228.

## Notes

Detailed composition of all simulated systems; force-field parametrization of cofactors and atomic mutagenesis variants; MD equilibration protocol and analysis; alchemical free-energy calculation setup and analysis; QM/MM simulation details including QM region definitions and free-energy barriers; *ab initio* p*K*_*a*_ calculations for deazaadenosine variants including regression model and benchmark tables; RMSD and RMSF analyses for all simulated systems; time series and joint distributions of key geometric descriptors; magnesium ion placement and 3D-RISM analysis; ProSeDMA derivatives and depurination pathway; examples of SAM-binding RNA motifs with divalent ions; hydrogen-bond contact tables for all cofactor systems (PDF)
