# Supplementary material for: Origins of Reactivity in SAM-Utilizing Ribozyme SAMURI-Catalyzed RNA Alkylation
Source: J Am Chem Soc. 2026 Jul 15;148(29):31316–30. doi: 10.1021/jacs.6c08579 (PMC13426253; doi:10.1021/jacs.6c08579)
Supplement: Supplementary file 1 [file ja6c08579_si_001.pdf]

Supporting Information: Origins of reactivity in SAM-utilizing ribozyme SAMURI-catalyzed RNA alkylation

Julie Puyo-Fourtine, Yanan Du, Erika McCarthy, Şölen Ekesan, and Darrin M. York*

Laboratory for Biomolecular Simulation Research, Institute for Quantitative Biomedicine and Department of Chemistry and Chemical Biology, Rutgers University, Piscataway, NJ 08854, USA

E-mail: Darrin.York@rutgers.edu

Data Availability Statement

Raw data, parameters, coordinates, input files, scripts, and trajectories related to the simulations performed in this study are available at https://zenodo.org/records/20027228

Detailed Computational Methods

Detailed composition of simulated systems

Table S1: Details of all systems builds including modifications and solvent components

Cofactor	Modification	Water	Na	Cl	Mg
MD and QM/MM					
SAM		10,874	72	28	6
SAM	$+Mg^{2+}$	11,075	70	28	7
SAM	dcAdoMet	11,139	72	29	6
ProSeDMA		12,173	79	31	4
QM/MM					
SAM	$+Mg^{2+}$,c^7A52	11,075	70	28	7
ProSeDMA	c^7A52	12,173	79	31	4
ProSeDMA	c^1A52	12,173	79	31	4

Cofactors included S-adenosylmethionine (SAM), decarboxylated S-adenosylmethionine (dcAdoMet), and propargylic Se-2,6-diaminopurinribosyl-selenomethionineamide (ProSeDMA).$+Mg^{2+}$ indicates the addition of a Mg^{2+} ion coordinating the cofactor tail.

Non-standard residue parametrization

ProSeDMA was parameterized using a fragment based procedure within the AMBER framework.[1] In this protocol, the cofactor was divided into three components: the diaminopurine nucleobase, the ribose moiety and the side chain with selenium. Charges for the diaminopurine fragment were first derived by RESP fitting of electrostatic potentials computed with Gaussian.[2,3] Charges for the sugar fragment were taken directly from the corresponding ribose fragment available in AMBER, using the variant consistent with the desired pro-

tonation state for the O3′ hydroxyl group. The a tail with selenium was parameterized separately as an independent methyl capped fragment. Its geometry was optimized at the MP2/6-31G* level of theory prior to electrostatic-potential calculations and RESP charge derivation. This fragment includes the propargyl group with selenium together with the rest of the side chain attached to the ribose, so that the final charge distribution remained consistent over the whole substituent. The three fragments were then assembled into a single cofactor description. In addition, the atom type of C5′ was reverted from `CT` to simplify the parameterization.

An initial parameter set for the assembled cofactor was then generated. Because ProSeDMA contains selenium, this automatically generated parameter set required additional manual refinement. Initial selenium parameters were adapted from sulfur analogies and these terms were subsequently refined using MP2/6-31G* optimizations[4] performed on tripropylselenium and tripropylsulfonium model compounds. Comparison of these two reference systems was used to adjust the selenium-specific force-field terms and improve the description of the Se-containing part of the cofactor (Figure S1 and Table S2).

ProSeDMA parameters

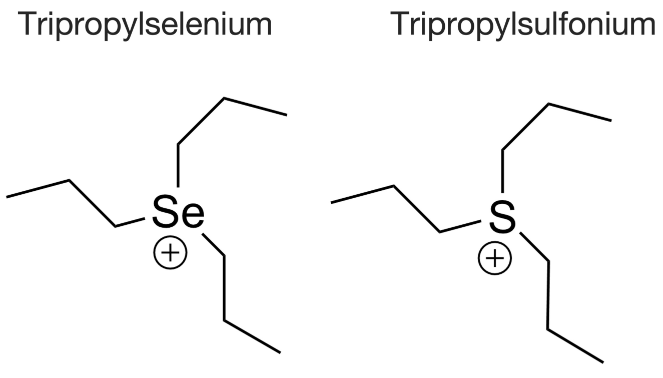

Figure S1: Representation of the tripropylselenonium and tripropylsulfonium model compounds used to derive the force-field parameters, highlighting the differences induced by replacing sulfur with selenium

Table S2: Comparison of force-field parameters for selenium (Se) and sulfur (S)

Parameter	Se	S
Mass (amu)	78.871	32.060
Bond length C3–X (Å)	1.952	1.831
Angle C3–X–C3 (deg)	99.493	96.120
Angle C1–C3–X (deg)	110.989	106.350
Angle C3–C3–X (deg)	111.530	110.120
Angle H1–C3–X (deg)	107.920	107.920
σ (Å)	2.180	2.000
ϵ ($\text{kcal} \cdot \text{mol}^{-1}$)	0.220	0.250
α (Å^3)	3.77	3.00

Atomic mutagenesis of the target adenosine

Because 1-deazaadenosine (c^1A) and 7-deazaadenosine (c^7A) are not standard AMBER residues, they were parameterized separately prior to system construction. Partial charges were obtained using the same Gaussian/RESP procedure as for the other nonstandard cofactors. Atom types were assigned from AMBER by selecting the closest possible analogies to those of native adenine, in order to reproduce the parent adenosine base as closely as possible.

Using the system with ProSeDMA, mutated variants were then introduced at the A52 base, where alkyl transfer takes place. For the c^1A and c^7A systems, the initial structures were generated directly from the equilibrated umbrella-sampling windows of the native adenosine (A) system. The mutations were introduced from the corresponding A structures in each of the 32 equilibrated umbrella-sampling windows to ensure consistency across all starting configurations.

MD simulation protocol

The system was equilibrated through a multistep protocol including solvent relaxation, gradual heating, density stabilization, and staged release of positional and restraints on atoms involved in the transfer reaction. Initially, global positional restraints were applied to all solute heavy atoms with a force constant of $k = 50\ \text{kcal} \cdot \text{mol}^{-1} \cdot \text{Å}^{-2}$.

In parallel, relevant restraints were applied using the same force constant. These included: (i) semi-harmonic restraints preserving the crystallographic Mg^{2+} crystal-packing ions, (ii) for SAM + Mg^{2+} system : a restraint on the Mg^{2+}–carboxylate contact (target distance 3.6 Å), (iii) a restraint on the NαH···O2(U8) hydrogen bond (target distance 2.63 Å), (iv) a semi-harmonic upper-bound restraint on the nucleophile–electrophile distance (boundary at 3.0 Å), (v) a semi-harmonic angular restraint enforcing an in-line attack geometry (lower bound 170°). For ProSeDMA system, additional restraints were introduced: (vi) to maintain the propargyl group of ProSeDMA in a π-stacking arrangement over the G10 base, three flat-bottom distance restraints were applied between the propargyl moiety and G10, keeping distances within the 3.2–4.2 Å range and preventing separation from the guanine surface; (vii) three upper-wall distance restraints were applied to preserve key interactions between G9, U37, and the cofactor, namely between G9(O6) and U37(O2′), G9(N1) and U37(O2), and ProSeDMA(N6) and U37(O4). These restraints were defined such that no penalty was applied below 3.2 Å, while a harmonic potential was applied beyond this distance. They were necessary to maintain the structural integrity of the structure, as their absence led to disruption of these interactions during the simulations.

This was followed by a procedure of progressive restraint reduction, as described below. An initial solvent minimization was performed while maintaining the positional restraints (k = 50), followed by three short NPT simulations at 300 K and 1 atm (5 ps, 5 ps, and 190 ps) to stabilize the box dimensions and density. The system was then re-minimized and heated under constant volume from 0 to 300 K over 600 ps, held at 300 K for 1 ns, and further equilibrated for 5 ns in the NPT ensemble. During all these preparation, heating, and early equilibration stages, both positional (`ntr`) and relevant restraints (`DISANG`) were maintained at $k = 50 \text{ kcal} \cdot \text{mol}^{-1} \cdot \text{Å}^{-2}$. Subsequently, the global positional restraints on the solute were progressively reduced through consecutive equilibration stages (25, 10, 5, and 2 $\text{kcal} \cdot \text{mol}^{-1} \cdot \text{Å}^{-2}$, while all restraints remained fixed at $k = 50$. Once positional restraints were fully removed (`ntr = 0`), an extended equilibration phase was carried out

in which only the relevant restraints were retained. A first 50 ns segment was performed with $k = 40$, followed by successive 10 ns segments in which the force constants associated with the defined restraints were progressively reduced to 30, 20, and 10 $\mathrm{kcal \cdot mol^{-1} \cdot \AA^{-2}}$. And then a last 5 ns segment with $k = 5\ \mathrm{kcal \cdot mol^{-1} \cdot \AA^{-2}}$ was performed. This gradual release allowed controlled relaxation of the hydrogen bond and in-line attack restraints while avoiding abrupt structural perturbations. For the systems with SAM, two Mg^{2+} ions from the crystallographic environment were retained even during production. These ions were relatively unstable during equilibration (drift close to the active site); therefore, restraints ($k \approx 40\ \mathrm{kcal \cdot mol^{-1} \cdot \AA^{-2}}$) were maintained on both crystallographic Mg^{2+} ions throughout production to preserve their crystallographic positions. In contrast, for the ProSeDMA systems, the crystallographic packing Mg^{2+} ion remained structurally stable during equilibration. Consequently, the restraints applied to the crystallographic Mg^{2+} ions were progressively reduced following the staged protocol described above, allowing gradual relaxation. However, the restraints between residues G9 and U37, as well as between ProSeDMA and U37, were maintained with a force constant of 30 $\mathrm{kcal \cdot mol^{-1}}$.

From these classical simulations, representative equilibrated structures were selected as starting points for subsequent QM/MM investigations of the adenine N3-alkylation reaction based on a catalytic fitness score.

Analysis of catalytic fitness

Catalytic fitness of the simulated ensembles was evaluated by monitoring the interaction between the nucleophile and electrophile. Two descriptors were used: (i) the distance between the alkyl carbon of the transferable group (methyl or propargyl) and the nucleophilic N3 atom of adenine, and (ii) the angle defined by the donor heteroatom (S or Se), the alkyl carbon, and N3. For each trajectory frame, a normalized geometric score was computed as

$$\mathrm{score} = \frac{\mathrm{value} - \mathrm{cutoff}}{\mathrm{best} - \mathrm{cutoff}}, \tag{1}$$

$$\text{combined score} = \sqrt{\frac{(\text{angle score})^2 + (\text{distance score})^2}{2}}. \quad (2)$$

For SAM-like and ProSeDMA-like cofactors best distance was set to 3.0 Å with a cutoff of 3.5 Å and the optimal angle was set to 170° with a cutoff of 140°. Frames below cutoff were assigned a score of zero. The resulting values ranged from 0 to 1 providing a direct measure of proximity to a catalytically relevant alignment.

Binding free energy calculations

Equilibration of the real state

After a first minimization, an equilibration phase of about 1 ns in the NPT ensemble is performed to allow adjustment of the simulation box volume, the temperature control is ensured by a Langevin thermostat[5] with a friction coefficient of 2 ps^{-1}, whereas the pressure is regulated with Monte Carlo barostat.[6] Once stabilized, a simulation in the NVT ensemble is following during 500 ps. The main equilibration phase of the real state consists in molecular dynamics of about 2 ns. A heating and cooling cycle is then applied taking the system from 300 K to 600 K, maintaining this elevated temperature and then returning it to 300. This aims to improve conformational diversity within the replicas. In this process of equilibration of the real state, the time step used is 1 fs, and the cutoff for the long range interactions is 10 Å.

Alchemical transformation

Simulations were performed using an optimized alchemical transformation pathway with second-order smoothstep softcore potential and 36-windows optimized phase space overlap λ schedule.[7] For the reference system: $\lambda \in$ [0.000000, 0.100340, 0.182850, 0.224850 0.260250, 0.290780, 0.316950, 0.340330, 0.357760, 0.369870, 0.380700, 0.392860, 0.409240, 0.428010, 0.446350, 0.465110, 0.483390, 0.499220, 0.513990, 0.529750, 0.546590, 0.563260, 0.578520,

0.593130, 0.609250, 0.628600, 0.649050, 0.665850, 0.681370, 0.700150, 0.727260, 0.754670, 0.783840, 0.820050, 0.899540, 1.000000]. For the RNA system: $\lambda \in$ [0.000000, 0.100510, 0.188890, 0.229220, 0.260910, 0.283890, 0.304900, 0.328100, 0.351030, 0.366140, 0.376250, 0.385310, 0.395760, 0.411070, 0.430620, 0.450040, 0.469780, 0.488820, 0.505410, 0.520690, 0.537010, 0.554650, 0.571780, 0.586870, 0.602200, 0.620460, 0.640160, 0.656310, 0.673330, 0.697510, 0.725370, 0.753600, 0.783220, 0.819860, 0.899440, 1.000000].

Each window was minimized and then equilibrated for 440 ps in the NPT ensemble, followed by 2 ns of equilibration with a heating cycle from 0 K to 300 K and then an NPT equilibration of about 8 ns. The production stage was then performed for 5 ns under NPT conditions. In this stage, hydrogen mass repartitioning was used, allowing a 4 fs timestep. A harmonic restraint centered at 2.10 Å between Mg^{2+}) and the phosphate oxygen was applied in a λ-dependent manner for the RNA and reference systems. This restraint was inactive at $\lambda = 0$ and smoothly activated as the particle is dummy ($k = 30\ \mathrm{kcal} \cdot \mathrm{mol}^{-1} \cdot \mathrm{\AA}^{-2}$). Window free energies were combined by MBAR[8,9] to obtain ΔG_{RNA} and ΔG_{ref}.

Analysis of alchemical free energy simulations

A

RNA

Decomposition of <ΔG> into trials				
Sampling	<ΔG>	ΔG#1	ΔG#2	ΔG#3
	453.30 ± 0.44	453.18 ± 0.60	452.61 ± 0.05	454.12 ± 0.06

B

Reference

Decomposition of <ΔG> into trials				
Sampling	<ΔG>	ΔG#1	ΔG#2	ΔG#3
	443.60 ± 0.40	443.34 ± 0.50	444.32 ± 0.04	443.01 ± 0.05

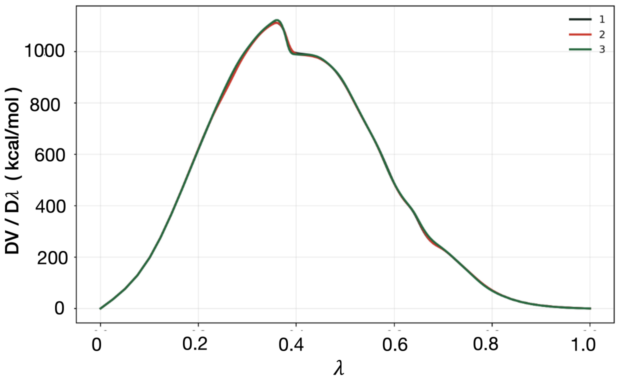

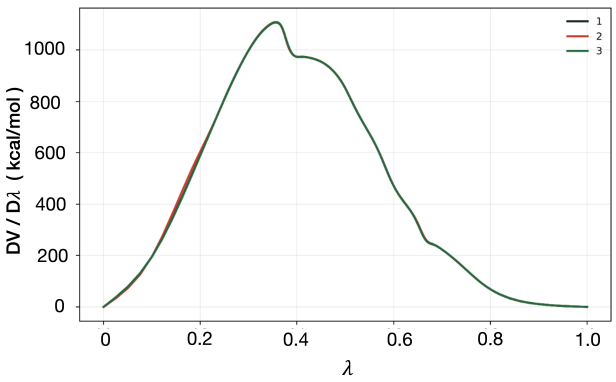

Figure S2: Analysis of alchemical free energy simulations for ion decoupling from two distinct environments: the RNA-SAM complex (A) and the phosphate reference system (B). Average $\frac{dU}{d\lambda}$ profiles obtained by MBAR[8] over three independent trials are shown. The cubic spline fit was integrated to determine the free energy of ion decoupling in each system.

Uncertainties in the MBAR free energy estimates were obtained using a circular moving block bootstrap procedure. Standard errors for free energy differences were then calculated by quadratic propagation of the uncertainties associated with the individual state free energies.[10]

Quantum Mechanics (QM)/Molecular Mechanics (MM) Simulations

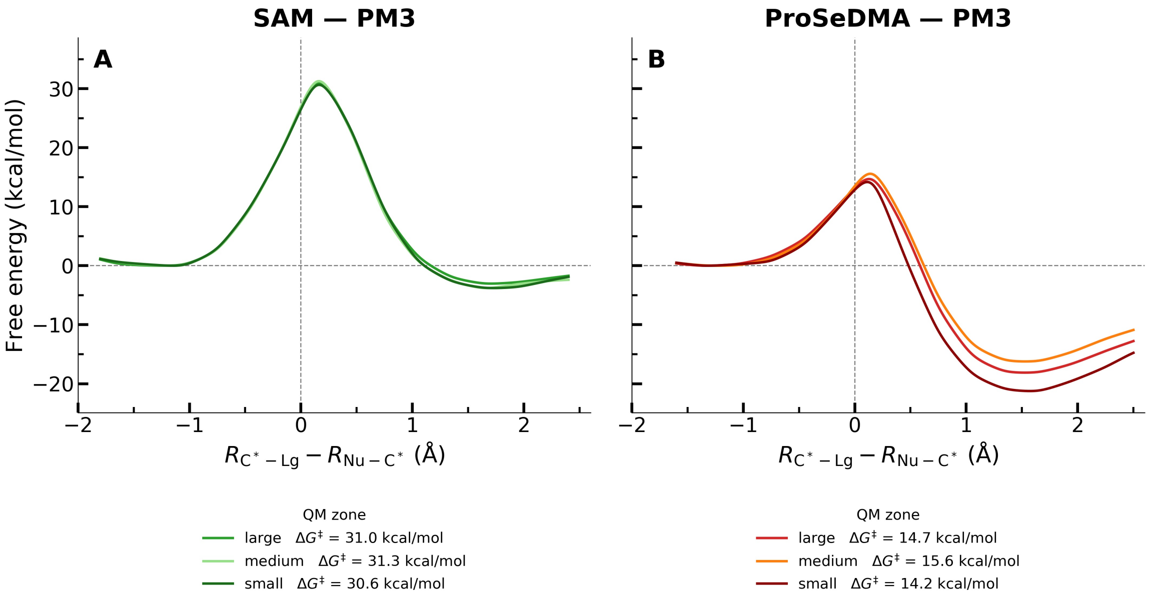

Figure S3: Benchmark of the QM region size for the free energy profiles of SAM and ProSeDMA at the PM3 level. Three QM region definitions were tested: *large*, including the entire ligand; *medium*, including the end of the tail with the electrophilic S or Se center and the entire sugar, with a cut at the glycosidic bond; and *small*, corresponding to the model used in this work (Figure S4), including the transferred methyl or propargyl group, the electrophilic S or Se center, and either the end tail for SAM retaining the CH_2 group adjacent to the sulfur center or the two adjacent CH_2 groups for ProSeDMA. The resulting QM region sizes are 64, 50, and 37 atoms for the *large*, *medium*, and *small* SAM models, respectively, and 84, 68, and 42 atoms for the corresponding ProSeDMA models. In all cases, base A52 is included in the QM region for both SAM and ProSeDMA. For ProSeDMA, base G10 is also included to account for the important π-stacking interaction with the ligand.

Uncertainties are reported (Table S3) bootstrap standard errors obtained by combining within replica bootstrap resampling (`--nboot 50`) and ensemble averaging across replicas.[10,11]

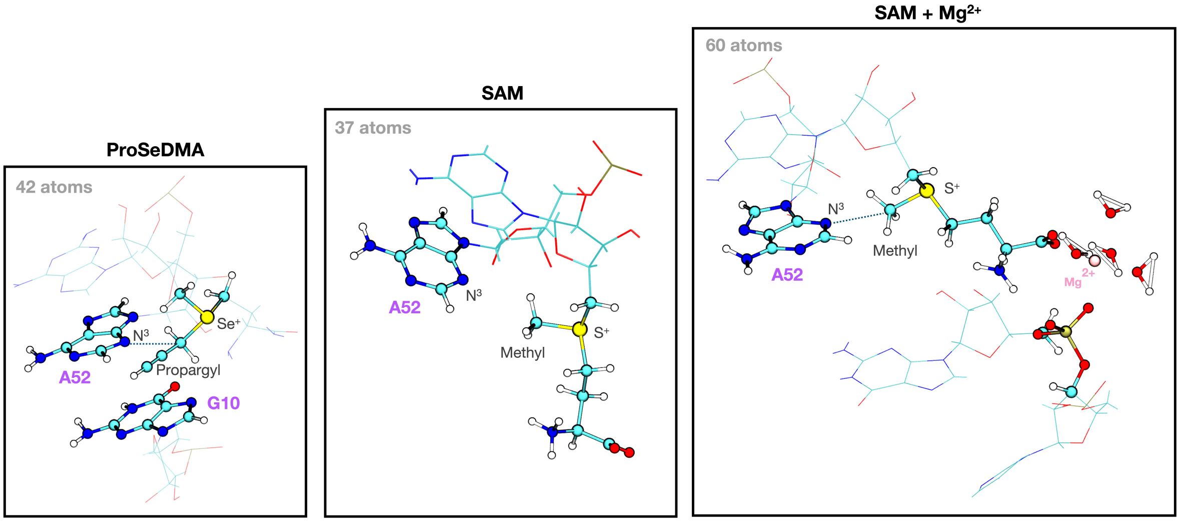

Figure S4: Representation of the different QM regions used (atoms shown with CPK representation) for the ProSeDMA (left), SAM (center), and SAM-Mg^{2+} (right) reactions.

Table S3: Free energy barriers and transition state uncertainties from QM/MM simulations.

System	$\Delta G^{\ddagger}$ (kcal mol^{-1})
ProSeDMA (A52)	21.9 ± 0.2
ProSeDMA-c^{1}A52	18.9 ± 0.1
ProSeDMA-c^{7}A52	17.8 ± 0.1
SAM	24.0 ± 0.1
SAM + Mg^{2+}	23.1 ± 0.1
SAM-c^{7}A52 + Mg^{2+}	20.2 ± 0.1

ab initio pK_a calculation

The c^{1}A:52 (1-deazaadenosine) and c^{7}A:52 (7-deazaadenosine) modifications were investigated by QM/MM simulations based on the hypothesis that replacing an endocyclic nitrogen, an electron withdrawing atom, with carbon would favor the localization of negative charge at the N3 position. This was expected to enhance nucleophilicity and, consequently, increase the pK_a. A recent study reported predicted pK_a values for related modified nucleobases,[12] but it underestimates the N3 pK_a of adenosine (0.8 vs. 1.5 experimentally[13]). We therefore recomputed these pK_a values and developed a more reliable predictive model leveraging available experimental data. To this end, a series of nitrogenous bases (Table S4) with experimentally measured pK_a values was selected and deprotonation free energies were calculated as described in the Computational Methods. These data were then used to fit a regression model for predicting experimentally unknown pK_a values.

In this study, the neutral form of adenosine (A) is defined as the N1, N3, and N7 deprotonated state, while the A:N1 is defined as the protonated form that carries a positive charge on the N1 site. To ensure consistency, reference compounds with the same protonation pattern—namely, neutral deprotonated structures and positively charged protonated states with known experimental pK_a values were selected as benchmarks for pK_a prediction, with results given in Table S4 Fitting Dataset. Previous studies have demonstrated that reliable pKa values can be obtained using methyl substituted nucleobase models, in which the ribose moiety is replaced by a methyl group.[12] This approach was therefore adopted in the present study to reduce computational cost.

Table S4: Regression model derived pK_as for benchmark modified nucleobases from *ab initio* DFT calculations at the M06-2X/aug-cc-pVTZ level of theory

Dataset	Compound	Position	pK_a^{calc}	pK_a^{expt}	pK_a^{model}	pK_a^{Error}
Fitting	A	N1	1.84	3.63	3.25	-0.38
	A	N3	0.28	1.50	2.21	0.71
	A	N7	-0.30	2.15	1.82	-0.33
	c^3A	N1	6.74	6.80	6.55	-0.25
	c^7A	N1	4.17	5.30	4.82	-0.48
	n^8A	N1	0.27	2.20	2.20	0.00
	m^6A	N1	3.29	4.01	4.23	0.22
	m^{66}A	N1	3.96	4.50	4.68	0.18
	m^7G	N1	8.21	7.20	7.53	0.33
						MAE: 0.32
						RMSE: 0.37
Testing	Purine	N1	0.81	2.40	2.56	0.16
	2AP	N1	2.73	3.80	3.85	0.05
	DAP	N1	4.22	5.10	4.85	-0.25
	C	N3	2.61	4.20	3.77	-0.43
	5nC	N3	-0.48	2.64	1.70	-0.94
	5mC	N3	3.36	4.30	4.28	-0.02
						MAE: 0.38
						RMSE: 0.43

pK_a values were calculated for nine different sites (Fitting Dataset) in derivatives of Adenosine (A), 3-deazaadenosine (c^3A), 7-deazaadenosine (c^7A), 8-azaadenosine (n^8A), 6-methyladenosine (m^6A), N6,N6-dimethyladenosine (m^{66}A), and N7-methylguanosine (m^7G) using M06-2X/aug-cc-pVTZ level of theory. A linear regression model ($y = 0.671x + 2.021$, with $R^2 = 0.9626$, where y represents the experimental pK_a values and x represents the calculated pK_a values) was constructed using the calculated (calc) and experimental (expt) pK_a values. Another set of pK_a values (Testing dataset) was calculated using the same level of theory for comprising the validation set of the regression model, including sites of Purine, 2-aminopurine (2AP), 2,6-diaminopurine (DAP), cytidine (C), 5-azacytidine (5nC) and 5-methylcytidine (5mC). p${K_a}^{\text{model}}$ denotes the pK_a values obtained from the linear regression model, p${K_a}^{\text{Error}}$ indicates the difference between the model derived and experimental pK_as (p${K_a}^{\text{model}}$ - p${K_a}^{\text{expt}}$). Mean absolute error (MAE) and root mean square error (RMSE) values were reported for both the fitting and testing datasets.

Geometry optimization and frequency calculations were performed at both the M06-2X/aug-cc-pVTZ[14,15] and the PBE0/6-31G*[16,17] levels of theory using the SMD implicit solvation model.[18] Tight convergence criteria and an ultrafine integration grid were employed throughout. Two linear regression models were constructed from these nine data points using the two levels of theory described above, as shown in Figure S5 and Figure S6, respectively.

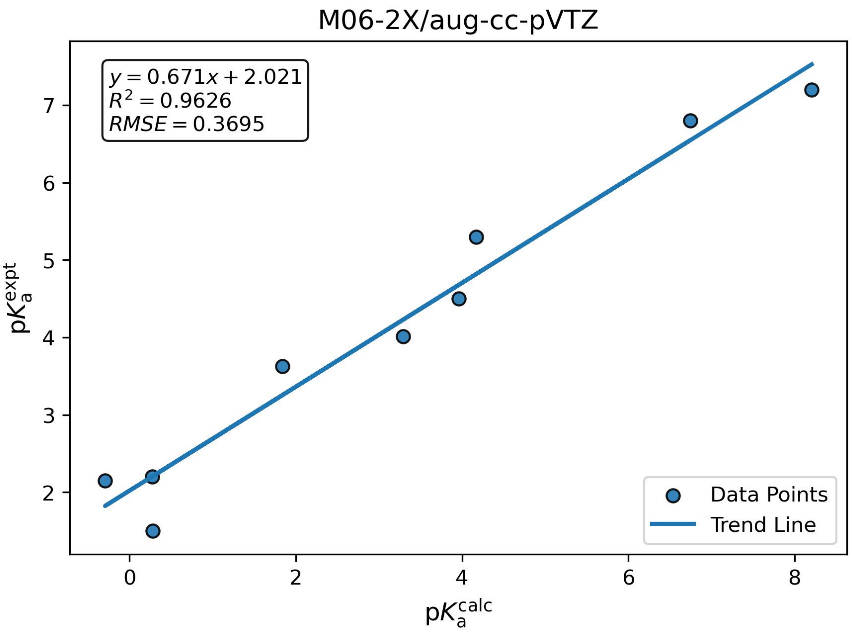

Figure S5: Experimental pK_a (pK_a^{expt}) vs. calculated pK_a (pK_a^{calc}) obtained using the M06-2X/aug-cc-pVTZ level of theory

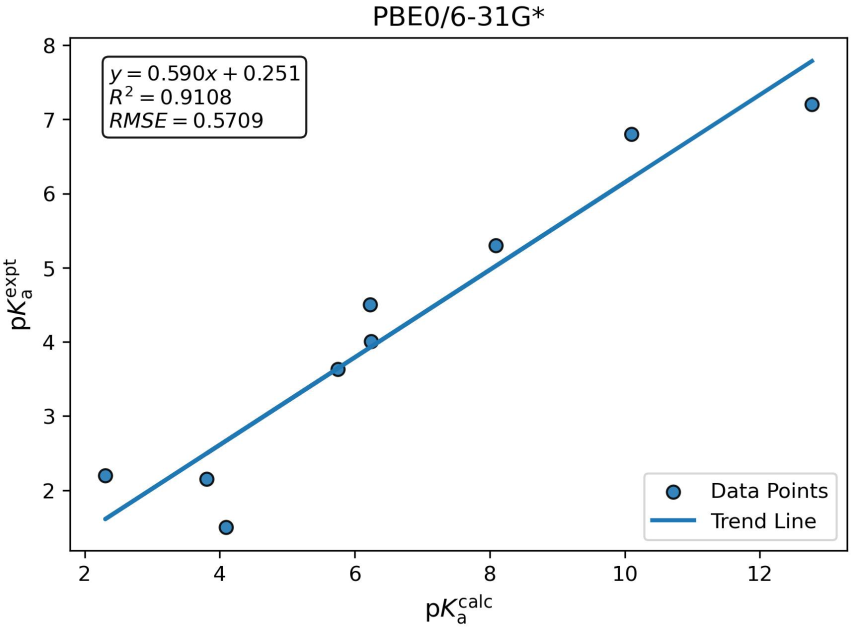

Figure S6: Experimental pK_a (pK_a^{expt}) vs. calculated pK_a (pK_a^{calc}) obtained using the PBE0/6-31G* level of theory.

Furthermore, the regression model based on the M06-2X/aug-cc-pVTZ level of theory was selected as the final model for estimating the pK_a values because it yielded better statistical

performance, with a higher R^2 value (0.9626 vs. 0.9108) and a lower RMSE (0.3695 vs. 0.5709). The resulting linear relationship is given by: $y = 0.671x + 2.021$, with $R^2 = 0.9626$, where y represents the experimental pK_a values and x represents the calculated pK_a values.

To further evaluate the predictive accuracy of this model, six additional compounds with known experimental pK_a values exhibiting analogous protonation patterns—specifically, neutral deprotonated and positively charged protonated states were examined, shown in Table S4 Testing Dataset. The same QM computational protocol was applied, and pK_a values were derived using the established regression equation. The model prediction errors pK_a^{Error}, defined as the difference between model derived and experimental pK_a values, were found to be less than 1 pK_a unit for all test dataset. MAE/RMSE values of 0.32/0.37 pKa units for the fitting set and 0.38/0.43 pKa units for the testing set indicate good reliability and transferability of the model.

With the validated function from our model, the model derived pK_a values at the N3 site are 5.20, 4.03, and 2.21 for c^1A, c^7A, and A respectively. Since the pK_a shift at the same site across different structures is expected to be minimal from a chemical standpoint, here the N3 site of A was applied to as a reference to predict the pK_a values of c^1A:N3 and c^7A:N3. The difference between the model derived and experimental pK_a for A:N3 (0.71, shown in Table S4) was hence considered as a systematic shift of the regression model to predict the pK_a values at N3 site, yielding predict pK_a values of 4.49 and 3.32 for c^1A:N3 and c^7A:N3, respectively(Equation 3).

$$\begin{aligned} \mathrm{p}K_a^{\text{pred}}{}_{\text{X:N3}} &= \left[\mathrm{p}K_a^{\text{model}}{}_{\text{X:N3}} - \mathrm{p}K_a^{\text{model}}{}_{\text{A:N3}}\right] + \mathrm{p}K_a^{\text{expt}}{}_{\text{A:N3}} \\ &= \mathrm{p}K_a^{\text{model}}{}_{\text{X:N3}} - 0.71 \end{aligned} \tag{3}$$

To assess the sensitivity of pK_a values to the level of theory, the same workflow was applied using the regression model at the PBE0/6-31G* level of theory (Figure S6). Specifically, the calculated pK_a values of 8.14 and 6.54 for c^1A:N3 and c^7A:N3, respectively, were

substituted into the linear regression equation derived from the PBE0/6-31G*/SMD level of theory ($y = 0.590x + 0.251$). The resulting values were subsequently applied to Equation 3, yielding final pK_a values of 3.89 and 2.94 for c^1A:N3 and c^7A:N3, respectively. These values follow the same overall trend as those obtained using the M06-2X/aug-cc-pVTZ model (Table S5).

Table S5: pK_a values at the M06-2X/aug-cc-pVTZ and PBE0/6-31G* levels of theory.

	M06-2X/aug-cc-pVTZ			PBE0/6-31G*		
Compound:Position	pK_a^{calc}	pK_a^{model}	pK_a^{pred}	pK_a^{calc}	pK_a^{model}	pK_a^{pred}
A:N3	0.28	2.21	–	4.09	2.67	–
c1A:N3	4.74	5.20	**4.49**	8.14	5.05	3.89
c7A:N3	2.99	4.03	**3.32**	6.54	4.11	2.94
c1c7A:N3	6.86	6.63	5.92	10.33	6.35	5.18

pK_a values are calculated (calc), model derived (model) and predicted (pred). The bolded pK_a values correspond to the final predicted pK_a values for c^1A:N3 and c^7A:N3. c1c7A:N3 denotes 1,7-dideazaadenosine on the N3 site, which is also calculated here, labeled in grey.

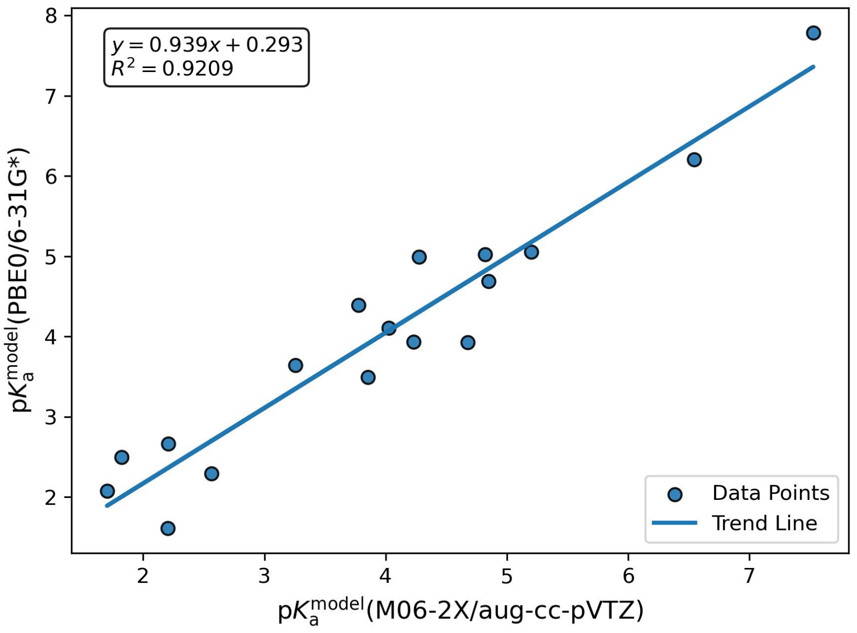

Figure S7: PBE0/6-31G* level of theory derived pK_a (p$K_a{}^{model}$) vs. M06-2X/aug-cc-pVTZ level of theory derived pK_a (p$K_a{}^{model}$) obtained using the corresponding linear regression model. All the examined data points, including c^1A:N3 and c^7A:N3, were included in the curve.

The resulting regression model obtained from PBE0/6-31G* level of theory showed somewhat reduced statistical performance relative to the M06-2X/aug-cc-pVTZ model (RMSE values of 0.57 and 0.37, respectively), but still exhibited a strong correlation with experimental pK_a values (R^2=0.91, as shown in Figure S6) and preserved the overall pK_a trends (Figure S7). Because the M06-2X/aug-cc-pVTZ model showed better agreement with the experimental calibration dataset, it was selected as the final model for pK_a estimation in this work, yielding predicted pK_a values of 4.49 and 3.32 for c^1A:N3 and c^7A:N3, respectively (Table S5). Despite the improved statistical performance of the higher level model, calculations at the PBE0/6-31G*/SMD level were substantially more computationally efficient (approximately 50-fold faster). Collectively, these results suggest that PBE0/6-31G*/SMD provides a computationally inexpensive approach that provides consistent overall pK_a trends to that of the higher level M06-2X/aug-cc-pVTZ/SMD model, although with somewhat reduced predictive accuracy.

Structure and dynamics of SAMURI in solution

Global stability of the solvated ribozyme

Root Mean Square Deviation (RMSD)

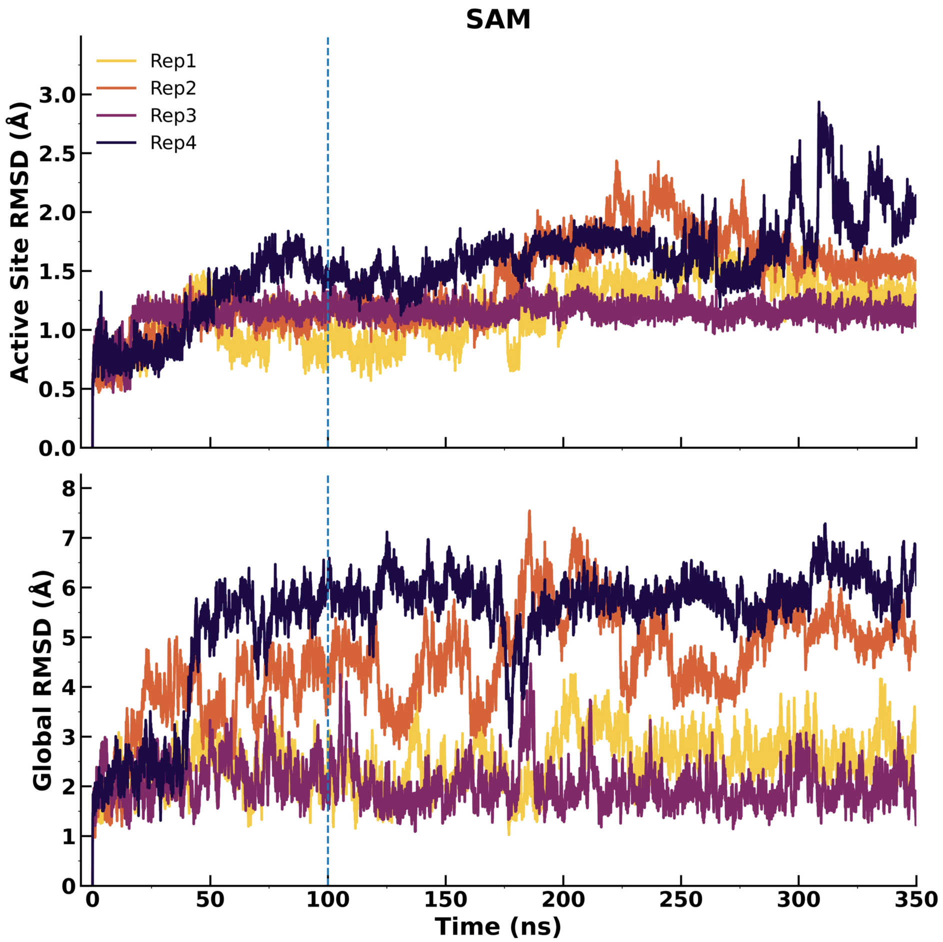

Figure S8: RMSD time series for the four replicas of SAM for the active site residues (residues 9–15, 30–37, 52 and 62) and the global system, across the four independent replicas. RMSD was calculated on all heavy atoms (non hydrogen atoms) using mass weighted fitting, with the first frame as reference.

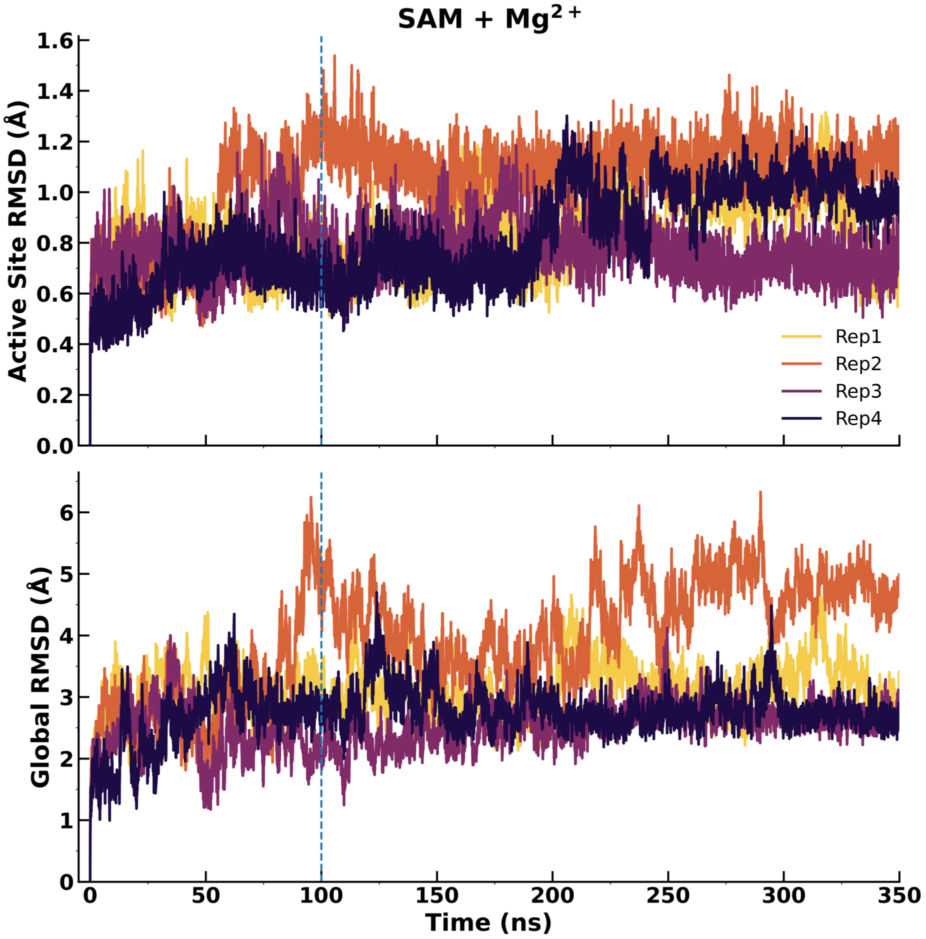

Figure S9: RMSD time series for the four replicas of SAM interacting with Mg^{2+} for the active site residues (residues 9–15, 30–37, 52 and 62) and the global system, across the four independent replicas. RMSD was calculated on all heavy atoms (non hydrogen atoms) using mass weighted fitting, with the first frame as reference.

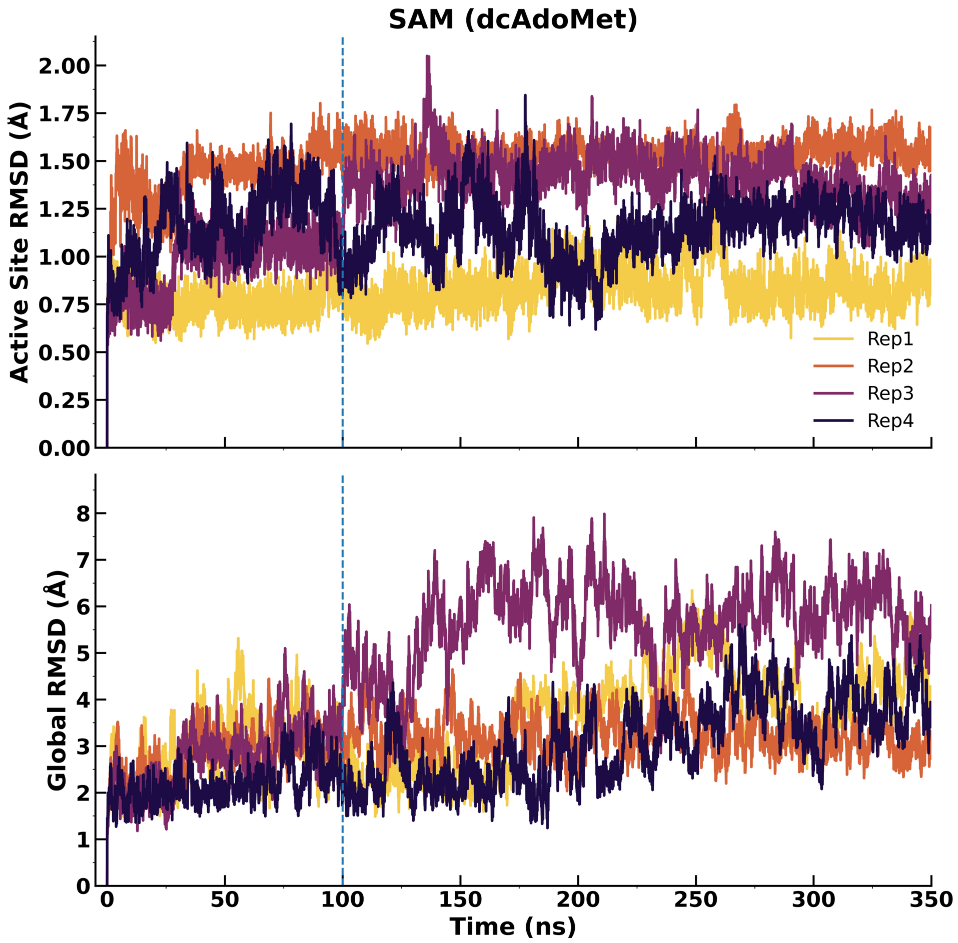

Figure S10: RMSD time series for the four replicas of SAM dcAdoMet for the active site residues (residues 9–15, 30–37, 52 and 62) and the global system, across the four independent replicas. RMSD was calculated on all heavy atoms (non hydrogen atoms) using mass weighted fitting, with the first frame as reference.

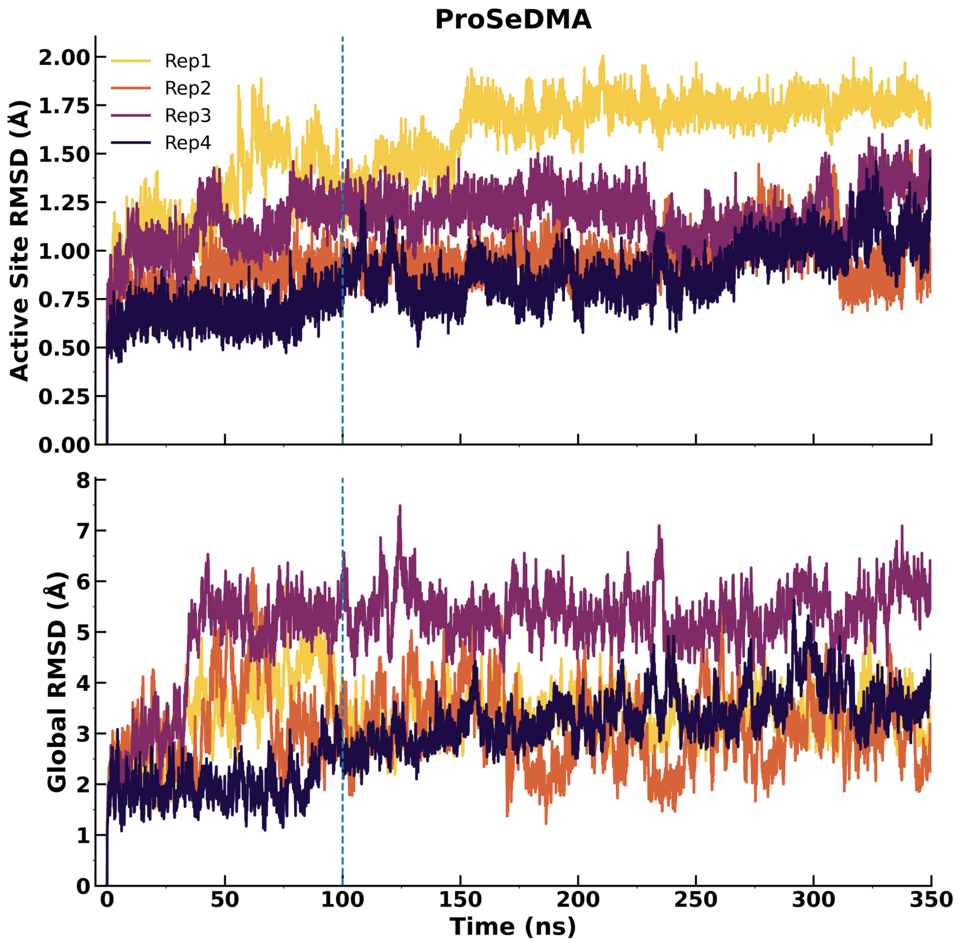

Figure S11: RMSD time series for the four replicas of ProSeDMA for the active site residues (residues 9–15, 30–37, 52 and 59) and the global system, across the four independent replicas. RMSD was calculated on all heavy atoms (non hydrogen atoms) using mass weighted fitting, with the first frame as reference.

Root Mean Square Fluctuation (RMSF)

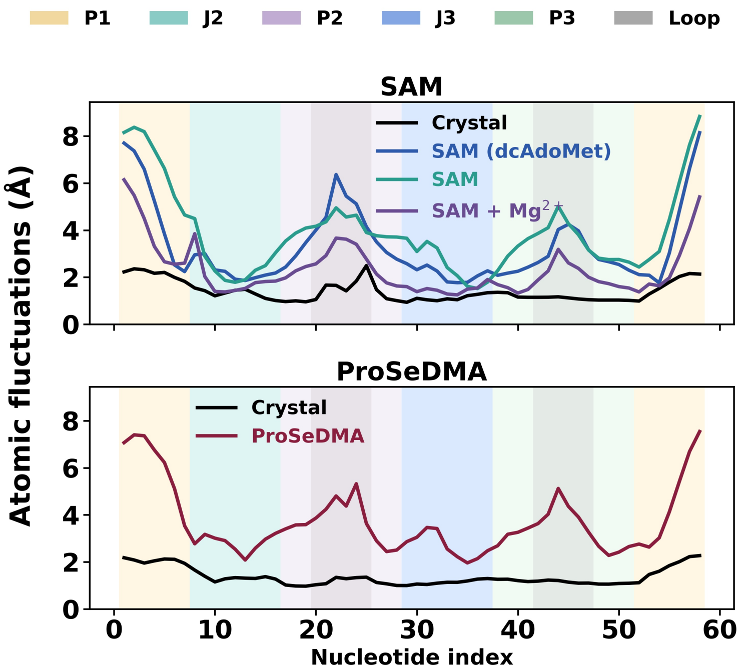

Figure S12: Root mean square atomic fluctuations (RMSF, Å) as a function of nucleotide index for the SAMURI ribozyme. Top: Systems containing SAM. The black curve represents the crystal structure. Fluctuations are shown for SAM dcAdoMet (blue), SAM (green), and SAM in the presence of Mg^{2+} (purple). Bottom: System containing ProSeDMA. The black curve represents the RMSF obtained from crystal structure B-factors and the burgundy curve corresponds to the ProSeDMA-bound form. Shaded regions indicate structural domains: P1, J2, P2, J3, P3 and the loop.

Time series and joint distributions for all simulated systems

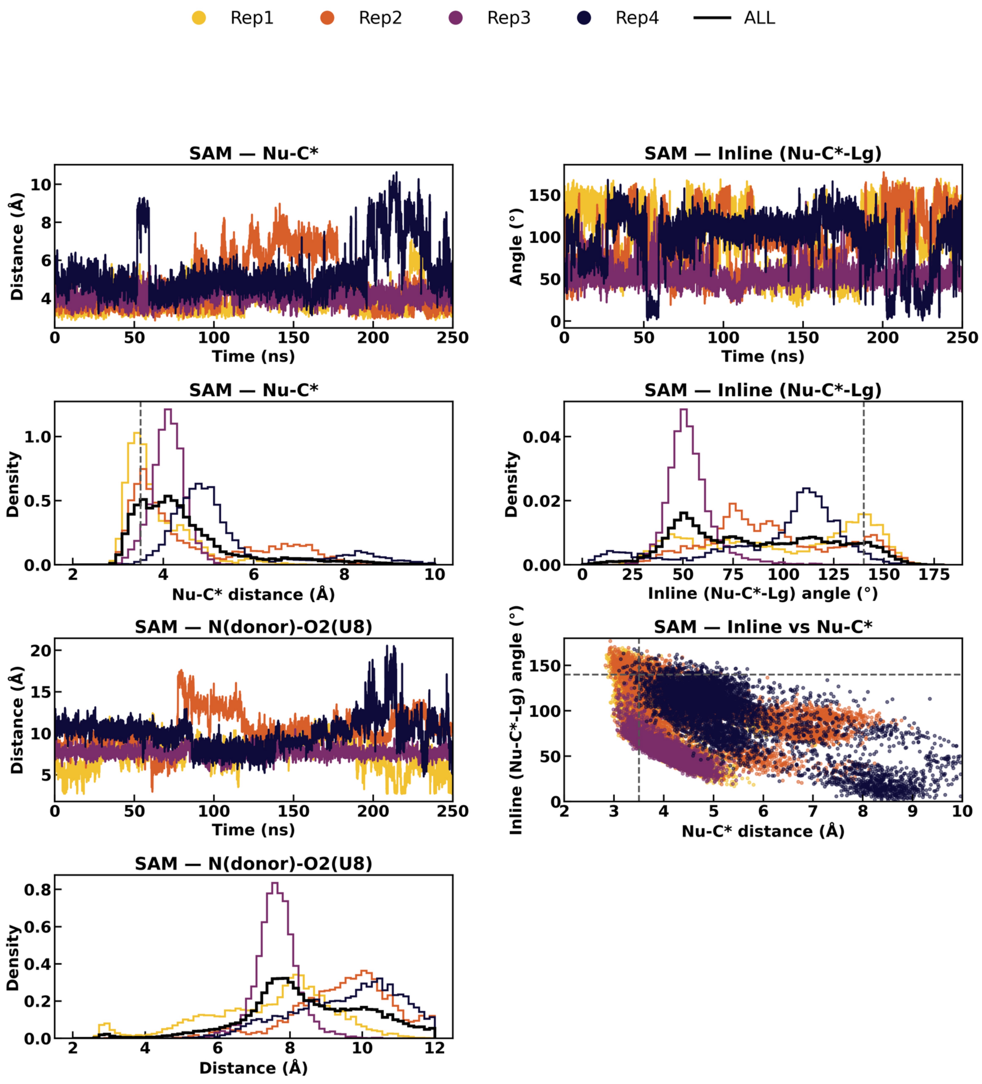

Figure S13: Time evolution of the Nu-C* distance, the inline attack angle (Nu-C*-Lg), and the N(donor)-O2(U8) distance, together with the joint distribution of the Nu-C* distance and inline angle, for the four independent 250 ns replicas of SAM.

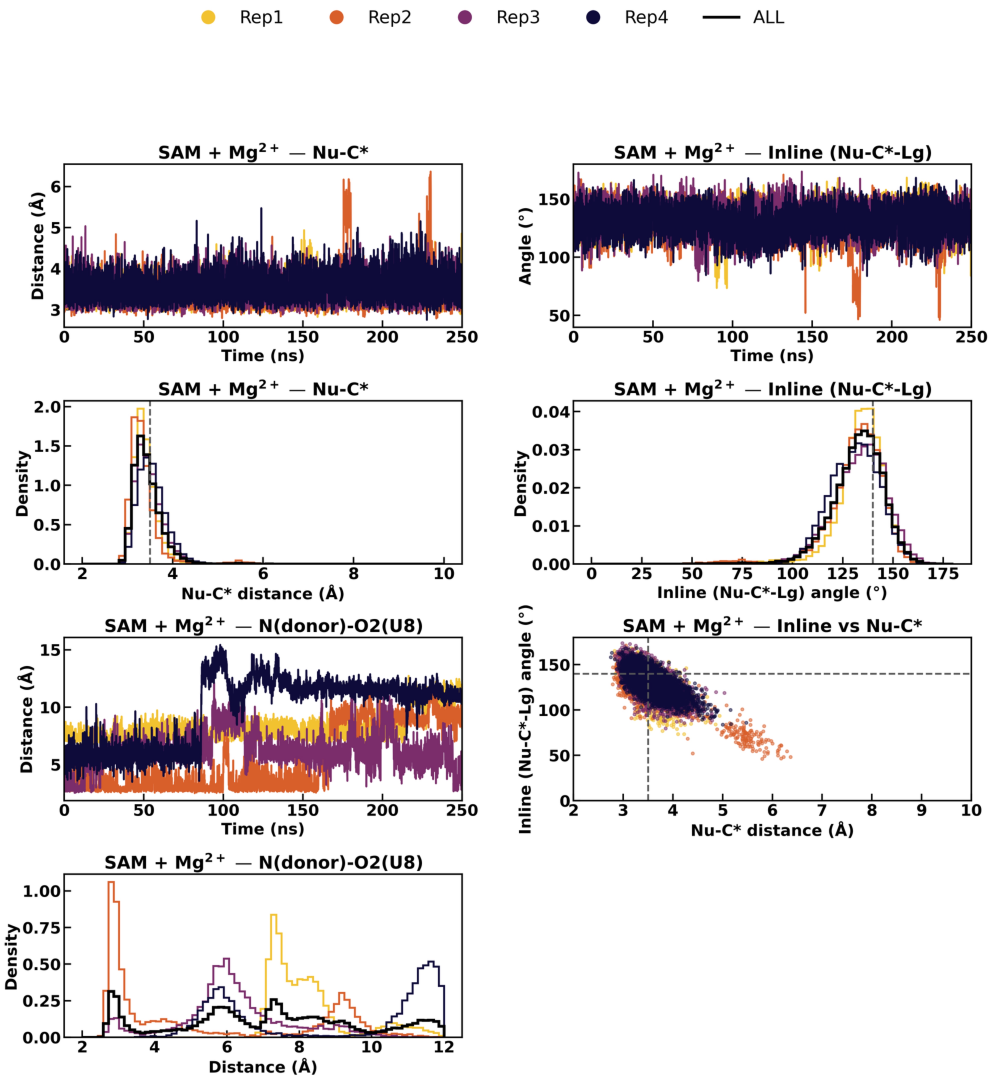

Figure S14: Time evolution of the Nu-C* distance, the inline attack angle (Nu-C*-Lg), and the N(donor)-O2(U8) distance, together with the joint distribution of the Nu-C* distance and inline angle, for the four independent 250 ns replicas of SAM in the presence of Mg^{2+}.

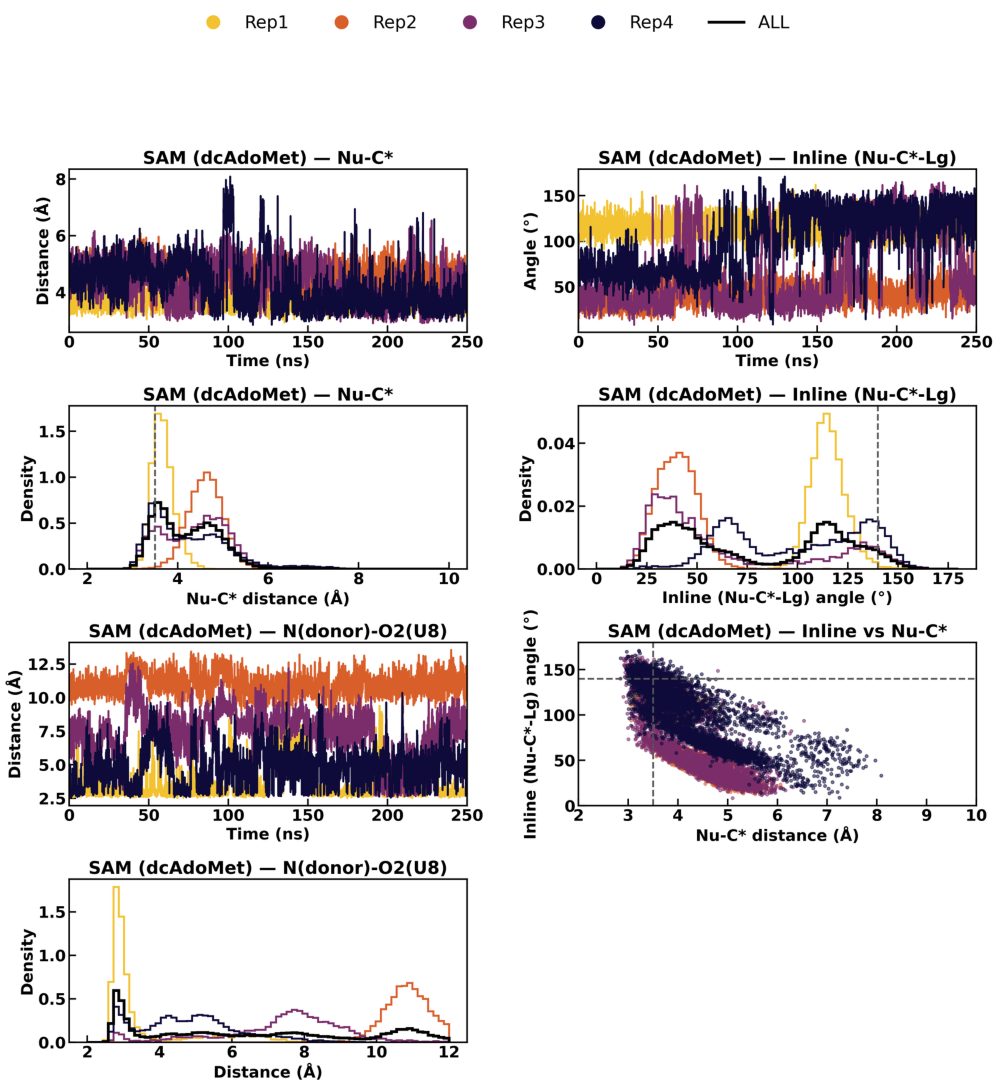

Figure S15: Time evolution of the Nu-C* distance, the inline attack angle (Nu-C*-Lg), and the N(donor)-O2(U8) distance, together with the joint distribution of the Nu-C* distance and inline angle, for the four independent 250 ns replicas of SAM (dcAdoMet).

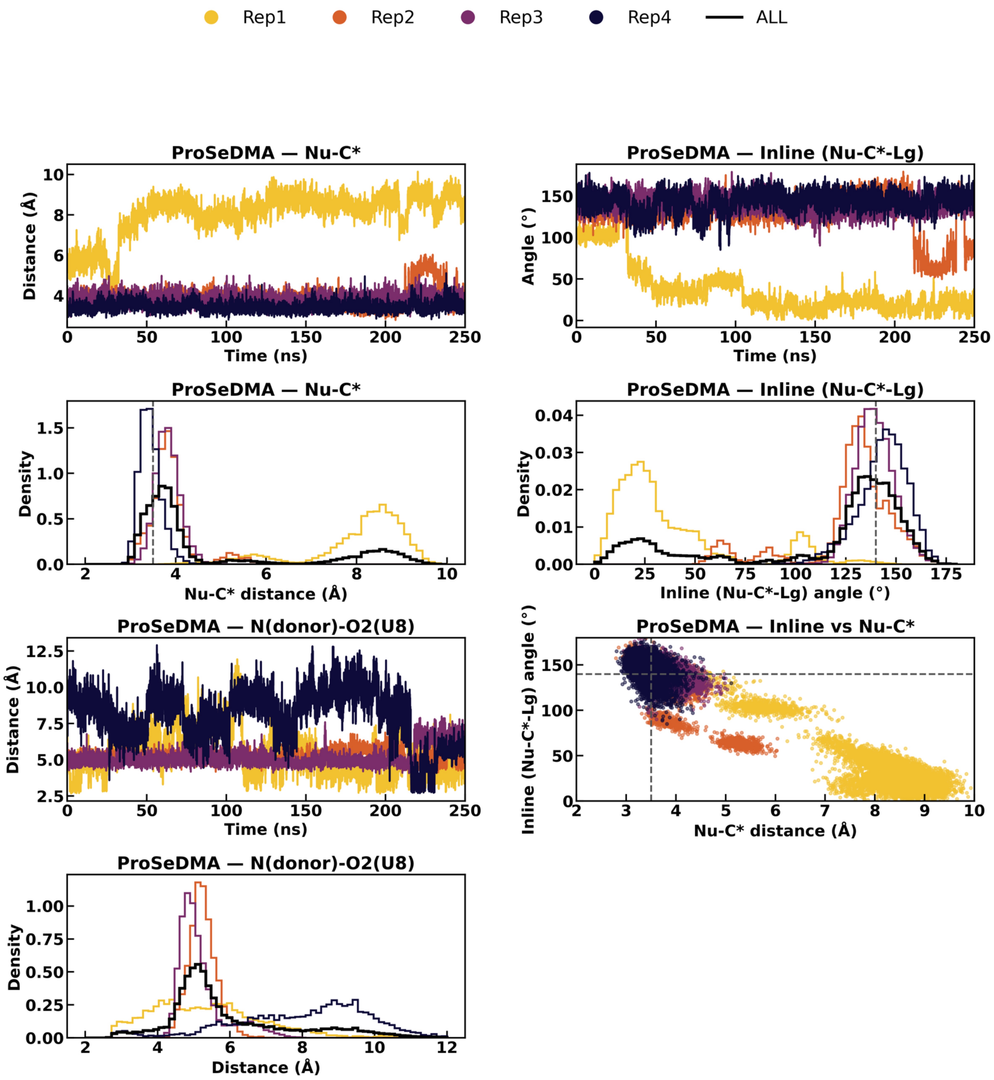

Figure S16: Time evolution of the Nu-C* distance, the inline attack angle (Nu-C*-Lg), and the N(donor)-O2(U8) distance, together with the joint distribution of the Nu-C* distance and inline angle, for the four independent 250 ns replicas of ProSeDMA.

Definition of the catalytically active state

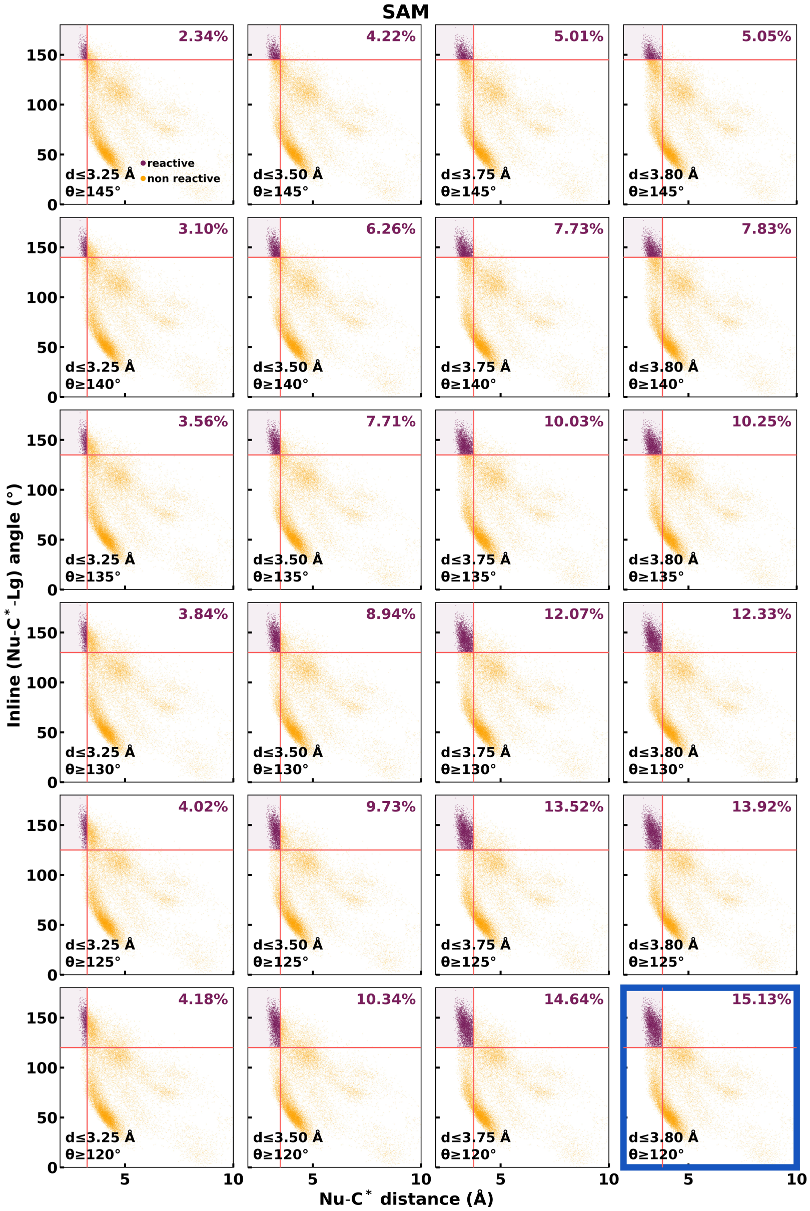

Figure S17: Sensitivity analysis of the reactive fraction for SAM from classical MD trajectories. Distributions of the in line attack angle $\angle(\mathrm{Nu-C^*-Lg})$ and the nucleophile–electrophile distance $R_{\mathrm{Nu-C^*}}$ are shown for different distance and angular cutoffs. The nucleophile (Nu) is A52:N3, C* is the electrophilic α-carbon of the cofactor and the leaving group (Lg) is the sulfur center (S). The fractions were computed from the analyzed portions of four independent trajectories, after discarding the first 100 ns of each 350 ns production run. Percentages shown in each panel report the fraction of frames satisfying the corresponding geometric criteria for the global ensemble. The blue box highlights the cutoff retained for the calculation of f_{react}.

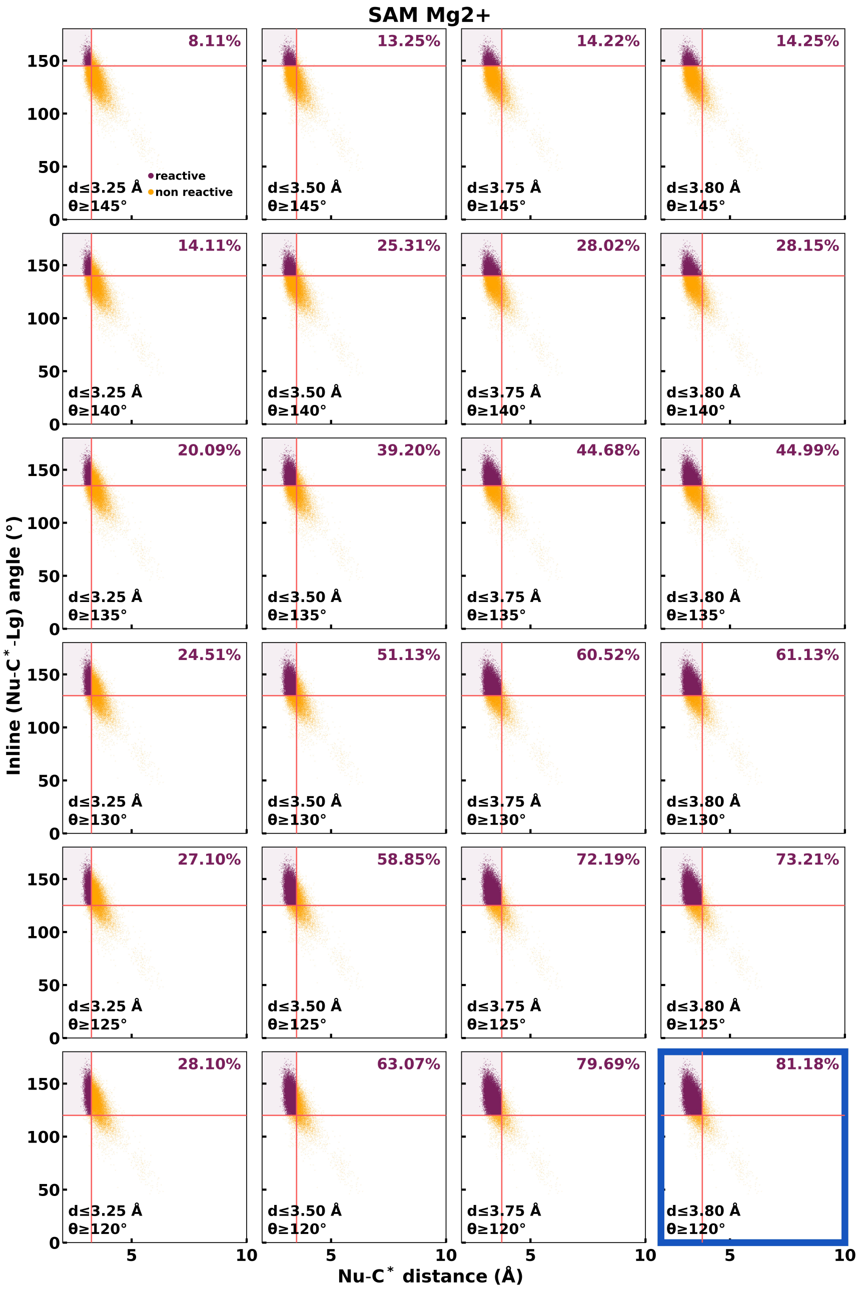

Figure S18: Sensitivity analysis of the reactive fraction for SAM + Mg^{2+} system from classical MD trajectories. Distributions of the in line attack angle $\angle(\mathrm{Nu}-\mathrm{C}^*-\mathrm{Lg})$ and the nucleophile–electrophile distance $R_{\mathrm{Nu}-\mathrm{C}^*}$ are shown for different distance and angular cutoffs. The nucleophile (Nu) is A52:N3, C^* is the electrophilic α-carbon of the cofactor and the leaving group (Lg) is the sulfur center (S). The fractions were computed from the analyzed portions of four independent trajectories, after discarding the first 100 ns of each 350 ns production run. Percentages shown in each panel report the fraction of frames satisfying the corresponding geometric criteria for the global ensemble. The blue box highlights the cutoff retained for the calculation of f_{react}.

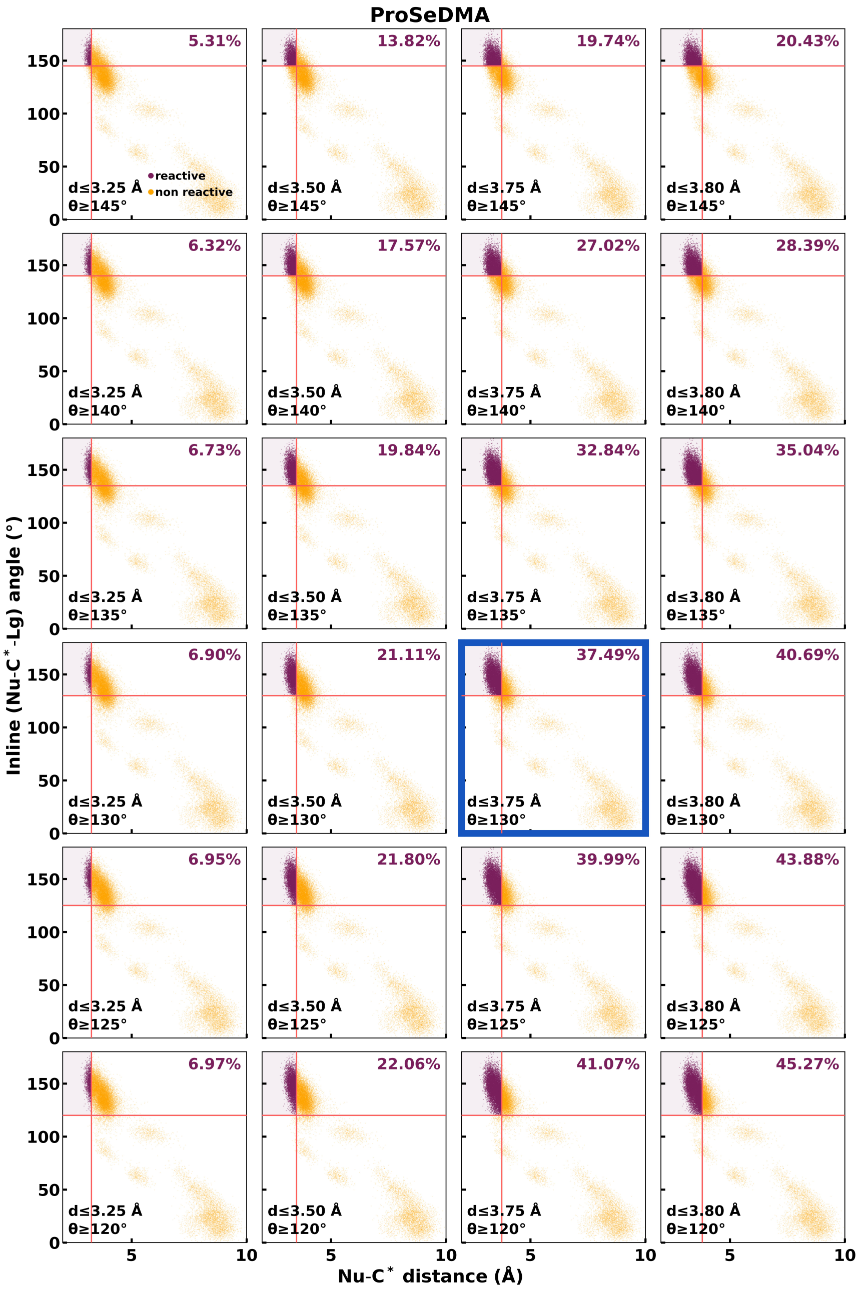

Figure S19: Sensitivity analysis of the reactive fraction for ProSeDMA from classical MD trajectories. Distributions of the in line attack angle $\angle(\mathrm{Nu} - \mathrm{C}^* - \mathrm{Lg})$ and the nucleophile–electrophile distance $R_{\mathrm{Nu-C^*}}$ are shown for different distance and angular cutoffs. The nucleophile (Nu) is A52:N3, C^* is the electrophilic α-carbon of the cofactor, and the leaving group (Lg) is the selenium center (Se). The fractions were computed from the analyzed portions of four independent trajectories, after discarding the first 100 ns of each 350 ns production run. Percentages shown in each panel report the fraction of frames satisfying the corresponding geometric criteria for the global ensemble. The blue box highlights the cutoff retained for the calculation of f_{react}.

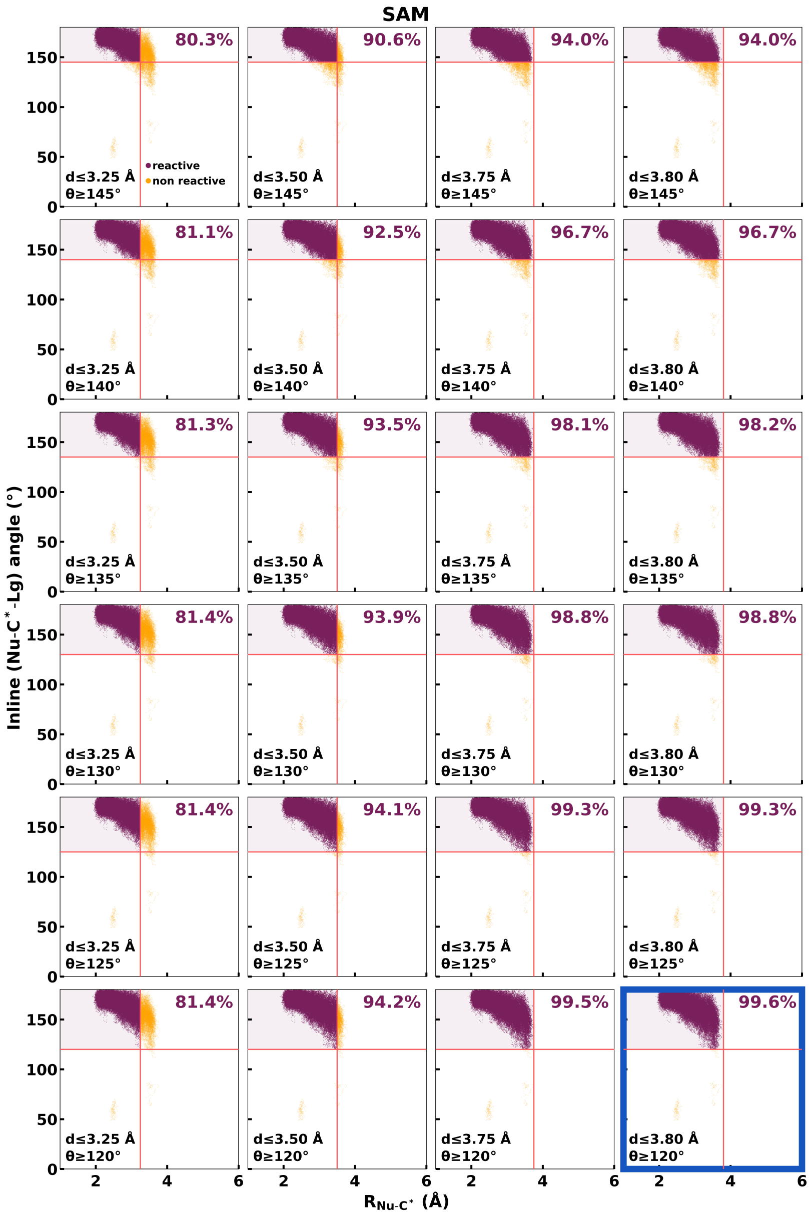

Figure S20: Sensitivity analysis of the geometric definition of the reactive ensemble for SAM using QM/MM umbrella sampling configurations up to the transition state region. Each panel shows the distribution of frames projected onto the nucleophile–C^* distance $R_{\mathrm{Nu-C^*}}$ and the inline attack angle $\angle(\mathrm{Nu}-\mathrm{C}^*-\mathrm{LG})$, for different distance and angular cutoffs. Purple points correspond to configurations satisfying both criteria, while orange points correspond to configurations outside the selected reactive region. The percentage indicates the fraction of QM/MM frames included within each cutoff definition. The blue box highlights the cutoff retained for the reactive ensemble definition.

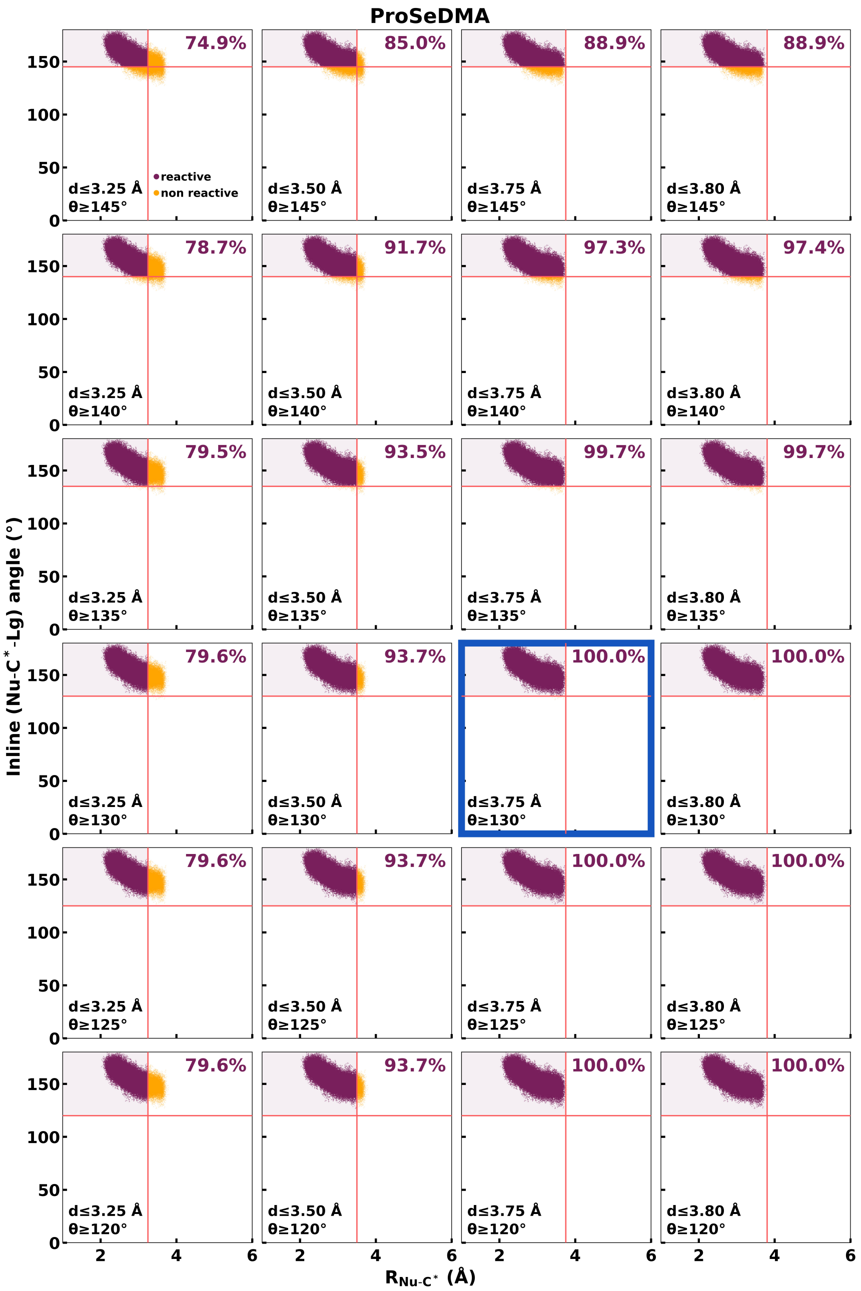

Figure S21: Sensitivity analysis of the geometric definition of the reactive ensemble for ProSeDMA using QM/MM umbrella sampling configurations up to the transition state region. Each panel shows the distribution of frames projected onto the nucleophile–C^* distance $R_{\mathrm{Nu-C^*}}$ and the inline attack angle $\angle(\mathrm{Nu-C^*-LG})$, for different distance and angular cutoffs. Purple points correspond to configurations satisfying both criteria, while orange points correspond to configurations outside the selected reactive region. The percentage indicates the fraction of QM/MM frames included within each cutoff definition. The blue box highlights the cutoff retained for the reactive ensemble definition.

Table S6: Sensitivity of f_{react} and ΔG_{react} to geometric cutoffs for SAM, ProSeDMA and SAM Mg^{2+}. f_{react}: fraction of reactive frames ($d \leq d_{\text{cut}}$, $\theta \geq \theta_{\text{cut}}$); $\Delta G_{\text{react}} = -RT\ln(f_{\text{react}})$, $T = 300$ K.

θ_{cut} (°) \ d_{cut} (Å)		SAM				SAM Mg^{2+}				ProSeDMA			
		**3.25**	**3.50**	**3.75**	**3.80**	**3.25**	**3.50**	**3.75**	**3.80**	**3.25**	**3.50**	**3.75**	**3.80**
**145**	f_{react} (%)	2.34	4.22	5.01	5.05	8.11	13.25	14.22	14.25	5.31	13.82	19.74	20.43
	ΔG_{react} (kcal mol^{-1})	2.24	1.89	1.79	1.78	1.50	1.20	1.16	1.16	1.75	1.18	0.97	0.95
**140**	f_{react}	3.10	6.26	7.73	7.83	14.11	25.31	28.02	28.15	6.32	17.57	27.02	28.39
	ΔG_{react}	2.07	1.65	1.53	1.52	1.17	0.82	0.76	0.76	1.65	1.04	0.78	0.75
**135**	f_{react}	3.56	7.71	10.03	10.25	20.09	39.20	44.68	44.99	6.73	19.84	32.84	35.04
	ΔG_{react}	1.99	1.53	1.37	1.36	0.96	0.56	0.48	0.48	1.61	0.96	0.66	0.63
**130**	f_{react}	3.84	8.94	12.07	12.33	24.51	51.13	60.52	61.13	6.90	21.11	**37.49**	40.69
	ΔG_{react}	1.94	1.44	1.26	1.25	0.84	0.40	0.30	0.29	1.59	0.93	**0.59**	0.54
**125**	f_{react}	4.02	9.73	13.52	13.92	27.10	58.85	72.19	73.21	6.95	21.80	39.99	43.88
	ΔG_{react}	1.92	1.39	1.19	1.18	0.78	0.32	0.19	0.19	1.59	0.91	0.55	0.49
**120**	f_{react}	4.18	10.34	14.64	**15.13**	28.10	63.07	79.69	**81.18**	6.97	22.06	41.07	45.27
	ΔG_{react}	1.89	1.35	1.15	**1.13**	0.76	0.28	0.14	**0.12**	1.59	0.90	0.53	0.47

To reduce the arbitrariness of the geometric definition, we used the complete set of QM/MM umbrella sampling frames up to the transition state. This provides a more objective definition of the reactive region, based on the ensemble of geometries actually sampled along the QM/MM reaction coordinate. For each QM/MM frame, we monitored the nucleophile–C^* distance, $r = R_{\mathrm{Nu-C^*}}$ and the attack angle, $\theta = \angle(\mathrm{LG} - \mathrm{C}^* - \mathrm{Nu})$. The frames were then projected onto the two dimensional (r, θ) space (Figures S20 and S21). A frame was classified as reactive when it satisfied both $r \leq r_{\mathrm{cut}}$ and $\theta \geq \theta_{\mathrm{cut}}$. The distance and angular cutoffs were varied to identify the saturation region that included nearly all configurations sampled in the QM/MM basin, corresponding to 99.6% of the SAM frames and 100% of the ProSeDMA frames. The same geometric definition was then applied unchanged to the classical MD trajectories to compute f_{react} (Figures S17 and S19). The corresponding free-energy contribution was calculated as

$$\Delta G_{\mathrm{react}} = -RT \ln(f_{\mathrm{react}}).$$

The resulting free energy corrections and intrinsic activation barriers are reported in Table S6. Overall, the absolute value of f_{react} varies with the selected distance and angular cutoffs, as expected. However, the corrected intrinsic barriers and the relative trends remain stable over the tested cutoff range, supporting the robustness of the analysis with respect to the reactive ensemble definition.

Table S7: Decomposition of the total free energy barrier $\Delta G^{\ddagger} = \Delta G_{\text{react}} + \Delta G^{\ddagger}_{\text{int}}$ for SAM and ProSeDMA systems. $\Delta G_{\text{react}} = -RT \ln(f_{\text{react}})$ at optimal geometric cutoffs; ΔG_{int}: intrinsic QM/MM barrier from umbrella sampling; $\Delta G^{\ddagger}_{\text{Expt}}$: experimental barrier derived from k_{obs}. All values in kcal mol^{-1}, T = 300 K.

**System**	ΔG_{react}	$\Delta G^{\ddagger}_{\text{int}}$	$\Delta G^{\ddagger}$
SAM	1.13	22.87	24.00
SAM+Mg^{2+}	0.12	23.01	23.13
SAM+c^{7}A52+Mg^{2+}	0.12	20.10	20.22
SAM (Expt.)	—		24.5
ProSeDMA	0.59	21.29	21.88
ProSeDMA+c^{1}A52	0.59	18.29	18.88
ProSeDMA+c^{7}A52	0.59	17.17	17.76
ProSeDMA (Expt.)	—		21.1

Comparison of geometric and harmonic models for the reactive fraction

Figure S22 shows the one dimensional probability distribution $P(r)$ along the nucleophilic attack distance $r = R_{\mathrm{Nu-C^*}}$ obtained from classical simulations, together with the corresponding free energy profile $-RT \ln P(r)$ for both SAM and ProSeDMA. The reactive population identified by the geometric criterion is also shown for comparison. Ideally, if the full conformational space could be sampled directly at the QM/MM level, including both reactive and unreactive conformations and their associated entropic contributions, this free energy term would naturally be included in the reaction coordinate itself. In that case, no separate definition of a reactive ensemble would be required. However, such exhaustive QM/MM sampling is not feasible given the timescales involved and the reactive fraction must therefore be estimated from classical simulations. To assess the sensitivity of this estimate to the definition of the reactive ensemble, we compared two approaches. The first is the geometric model used in the main analysis, which defines reactive frames based on both the nucleophilic attack distance and the inline attack angle. The second is a one dimensional distance based on the harmonic approximation, in which the reactive fraction is estimated from the probability of configurations falling within the reactive range along r only. In both cases, the corresponding free energy contribution was computed as $\Delta G_{\mathrm{react}} = -RT \ln(f_{\mathrm{react}})$, where f_{react} is the fraction of reactive frames. The distance based on the harmonic model should be viewed as an approximate description, since the Nu–C^* distance is a necessary but not sufficient condition for reactivity. A configuration may have a favorable attack distance while displaying an unfavorable leaving group–C^*–Nu angle, and therefore may not be properly preorganized for reaction. A contrario, the geometric criterion explicitly includes both the distance and angular requirements. As expected, the harmonic model yields systematically larger reactive fractions than the geometric model, because it includes a broader set of conformations along r without imposing the additional angular constraint. The corresponding values are 31.5% vs. 15.1% for SAM and 51.5% vs. 37.5% for ProSeDMA. Nevertheless, the resulting intrinsic activation free energy barriers ΔG_{Ref} remain very similar, differing

by only +0.4 kcal mol^{-1} for SAM and +0.2 kcal mol^{-1} for ProSeDMA. Thus, although the two definitions lead to different estimates of f_{react}, the resulting intrinsic barriers are nearly unchanged, supporting the robustness of our analysis with respect to the chosen definition of the reactive ensemble.

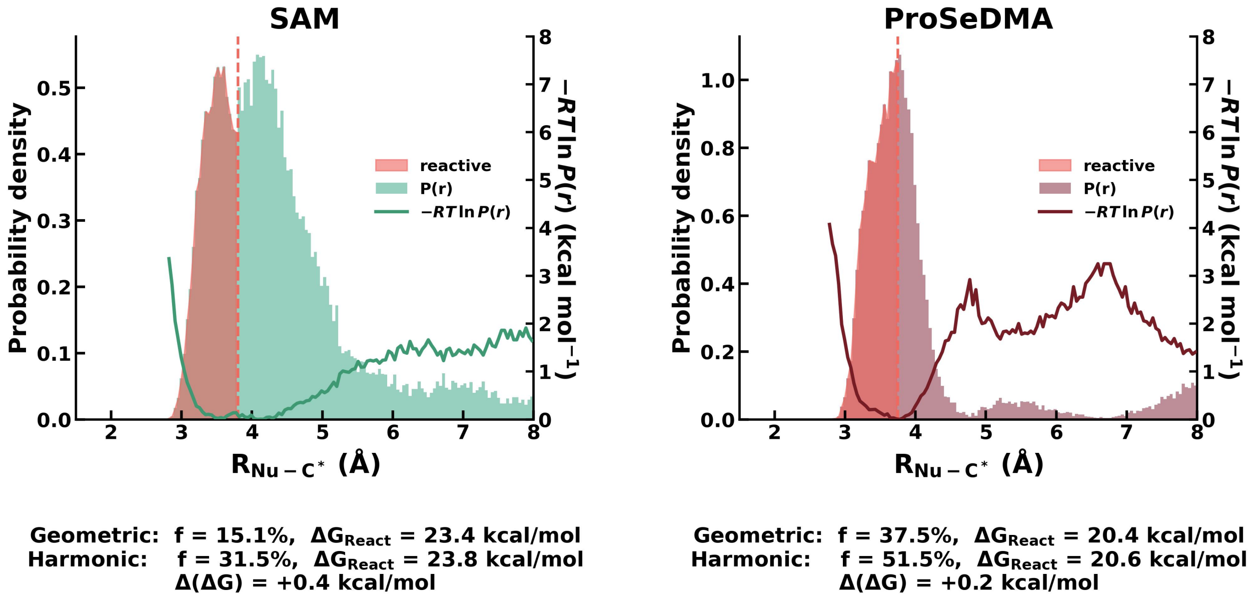

Figure S22: Assessment of the reactive fraction using a harmonic oscillator model along the nucleophilic attack distance $R_{\text{Nu}-\text{C}^*}$ for SAM and ProSeDMA, and comparison with the geometric model based on both distance and angle criteria (SAM : d $\leq 3.8\mathring{A}$ and $\theta \geq 120°$; ProSeDMA : d $\leq 3.75\mathring{A}$ and $\theta \geq 130°$)

Magnesium coordination in cofactor binding

In addition to the Mg^{2+} ion coordinated to the cofactor tail described in the main text (shown in orange in Figure S24A), we identified the need for a further divalent ion bridging the pro-R_P oxygens of residues U12 and C11 (highlighted in green Figure S24A). This site was first suggested by the accumulation of Na^+ ions at short distances in this region (Figure S24B) and was further supported by a pronounced Mg^{2+} density maximum in the 3D-RISM analysis (Figure S24C). The completed SAM model therefore contains seven Mg^{2+} ions in total.

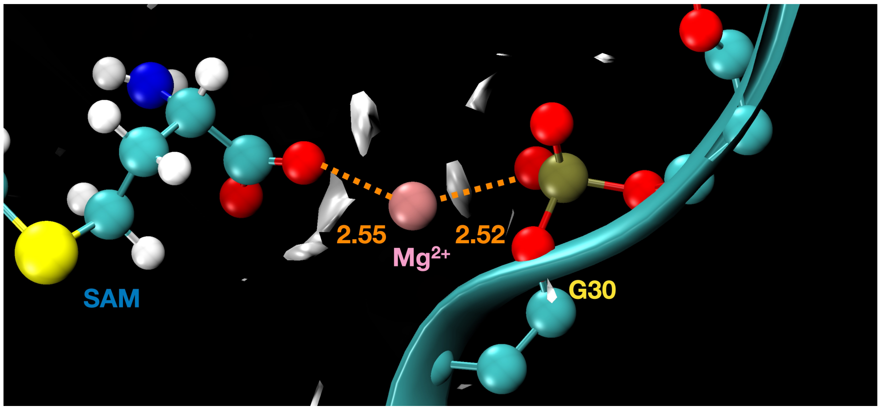

Figure S23: Prediction of a Mg^{2+} binding site from 3D-RISM from the crystal structure. A density maximum was identified between the SAM carboxylate and the G30 phosphate. A Mg^{2+} ion was therefore placed at ∼2.5 Å from the pro-S_P oxygen of G30 and from a SAM carboxylate oxygen. This chemically plausible starting geometry was subsequently refined during equilibration.

However, the structure with the ProSeDMA cofactor does not have a carboxylate group at the end of the cofactor and therefore does not have the additional Mg^{2+} observed in the SAM system. Nevertheless, 3D-RISM analysis revealed a comparable Mg^{2+} density between the pro-R_P oxygens of U12 and C11, and here too, a divalent ion was placed in direct coordination with these phosphates (shown in green in Figure S24A). The corresponding Mg–O distances are slightly longer (∼2.7 Å). The ProSeDMA model therefore contains four Mg^{2+} ions in total. Figures S24B and S24C highlight this location by comparing the Na^+ artifacts observed in the absence of Mg^{2+} with the corresponding Mg^{2+} density predicted by 3D-RISM.

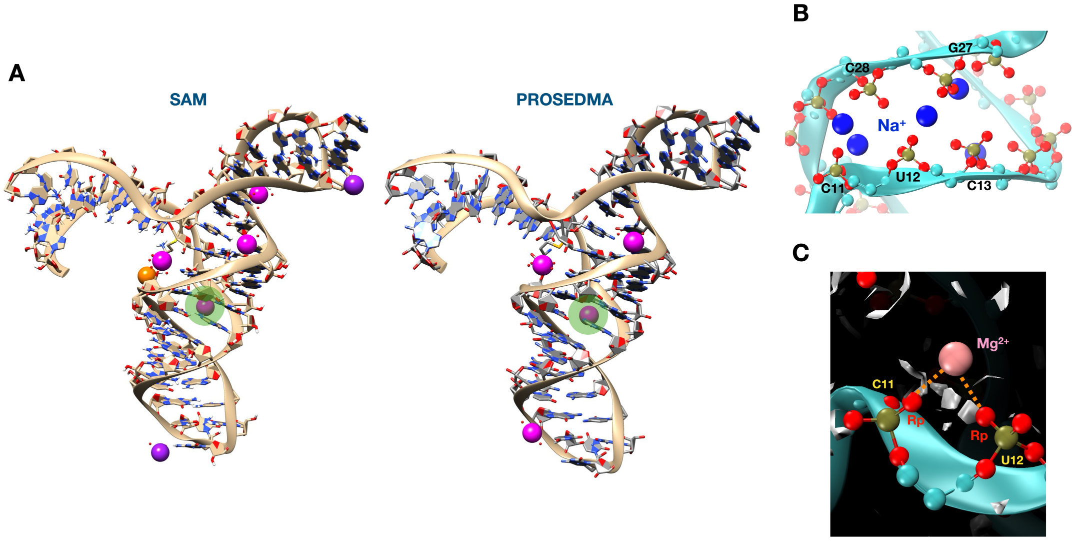

Figure S24: (A) Representations of SAMURI ribozyme with SAM (left) and ProSeDMA (right) cofactors, with Mg^{2+} ions shown as spheres. For SAM, two Mg^{2+} ions shown in purple correspond to those most likely associated with crystal packing. Because our simulations contain only a single subunit, these ions had to be restrained in order to be maintained at their crystallographic positions. The Mg^{2+} bound near the cofactor carboxylate in orange, and the additional bridging ion (U12–C11) in green. For ProSeDMA, the analogous bridging Mg^{2+} is shown in green. **(B)** Snapshot showing accumulation of Na^{+} ions of clustered negative charges in the absence of Mg^{2+}. **(C)** 3D-RISM prediction from the crystal of Mg^{2+} density, supporting placement of a bridging ion between pro-R_P oxygens of U12 and C11. Distances between Mg^{2+} and the pro-R_P oxygens are ~2.5 Å in SAM and ~2.7 Å in ProSeDMA.

ProSeDMA derivatives

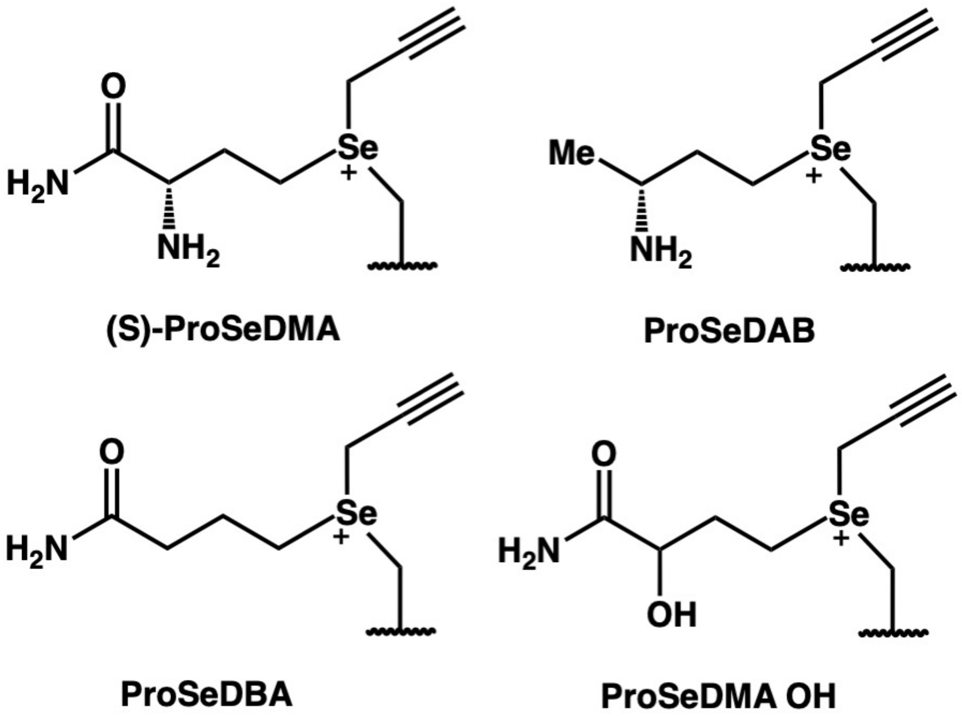

Figure S25: Chemical structure of ProSeDMA methionine unit and its derivatives mentioned in the main text. S-ProSeDMA: S-propargylic Se-2,6-diaminopurin-ribosyl-selenomethionineamide, ProSeDAB: Propargylic Se-2,6-Diaminopurineribosylseleno-2-(R)-amino-butane,ProSeDBA: Se-Propargyl-Se-2,6-Diaminopurineribosyl-selenobutanamide and ProSeDMA OH: Se-Propargyl-Se-2,6-diaminopurineribosyl-2-hydroxy-4-selenobutanamide.

Depurination pathway of c¹A variants

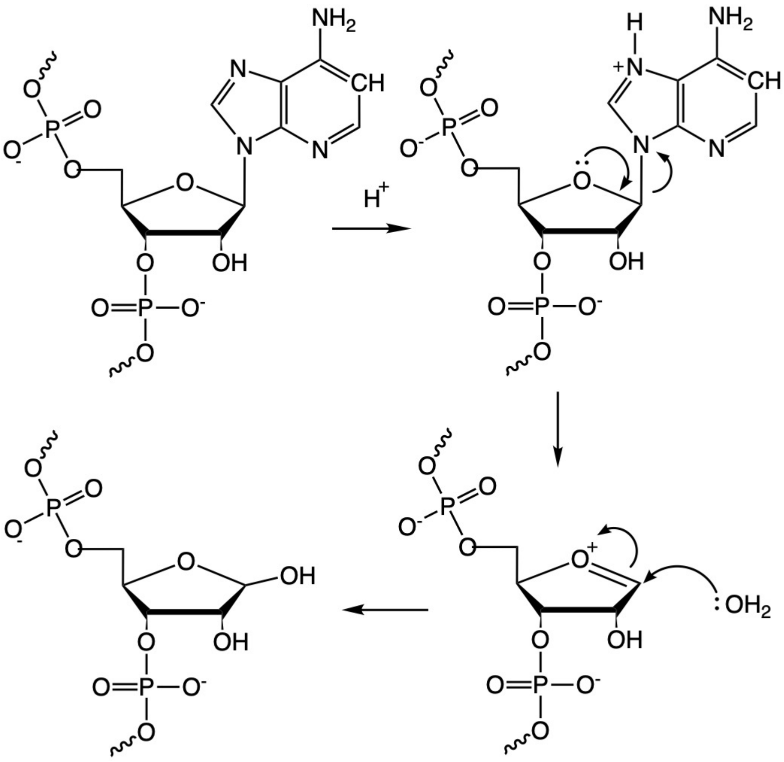

Figure S26: Depurination mechanism of adenosine resulting in the minor depurinated product for reactions of the c¹A modified ribozyme.

Examples of SAM-binding RNA motifs with divalent ions

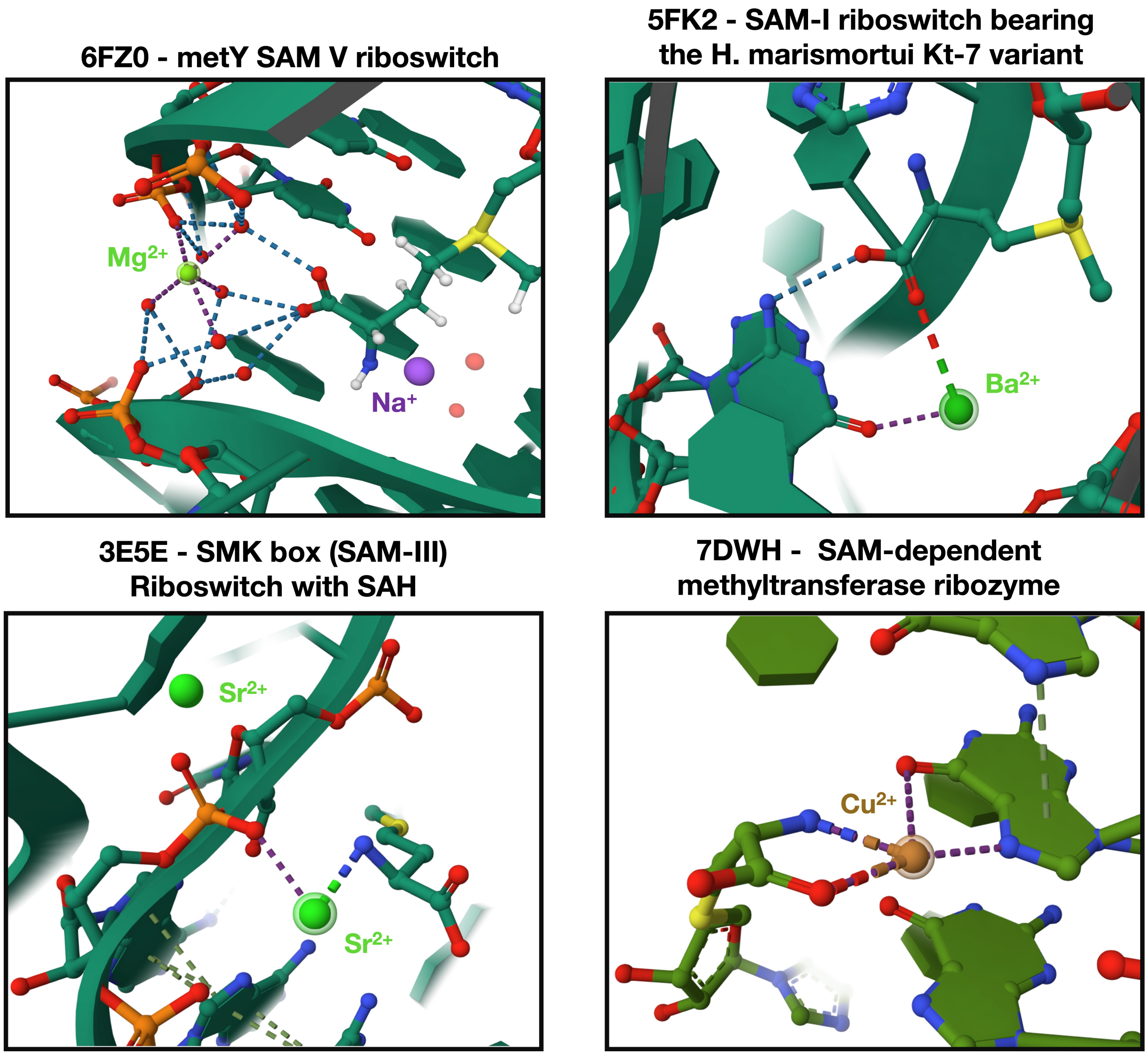

Figure S27: Representation of examples illustrating systems in which SAM acts as a cofactor interacting with divalent ions. The PDB codes are mentioned.[19–22]

Table S8: Main interaction partners of the cofactor amine nitrogen(s) in the different systems. Fractions indicate the proportion of frames in which the corresponding contact is observed. For ProSeDMA, the two amine nitrogens are reported separately; N05 corresponds to the Nα.

SAM

Atom	Partner	Fraction
:62@N1	G53@O5′	0.24
:62@N1	G53@OP2	0.13
:62@N1	G9@OP1	0.13
:62@N1	A7@N7	0.12
:62@N1	G10@OP2	0.09
:62@N1	G9@OP2	0.07
:62@N1	G53@O4′	0.04
:62@N1	G53@O6	0.04
:62@N1	A52@O3′	0.03
:62@N1	U8@OP2	0.02
:62@N1	G10@OP1	0.01
:62@N1	U5@O4	0.01

SAM +Mg^{2+}

Atom	Partner	Fraction
:62@N1	G10@OP1	0.69
:62@N1	A7@N7	0.31
:62@N1	A7@N1	0.17
:62@N1	G10@OP2	0.12
:62@N1	A7@N6	0.07
:62@N1	U8@O2	0.05
:62@N1	U8@O4	0.05
:62@N1	G53@O6	0.03

SAM (dcAdoMet)

Atom	Partner	Fraction
:62@N1	G10@OP1	0.22
:62@N1	G53@O5′	0.08
:62@N1	G53@O4′	0.08
:62@N1	G10@OP2	0.05
:62@N1	G53@OP2	0.05
:62@N1	U8@O2	0.04
:62@N1	A52@O3′	0.03
:62@N1	G53@O6	0.03
:62@N1	A7@N7	0.03
:62@N1	G10@O6	0.02
:62@N1	U8@O2′	0.01
:62@N1	G53@N7	0.01

ProSeDMA

Atom	Partner	Fraction
N05 (Nα)		
:52@N05	G53@O4′	0.28
:52@N05	G53@O5′	0.21
:52@N05	G10@N7	0.16
:52@N05	G9@O2′	0.10
:52@N05	U8@O2′	0.07
:52@N05	A52@O3′	0.07
:52@N05	G53@OP2	0.05
:52@N05	U8@O2	0.02
:52@N05	A52@N3	0.01
N01		
:52@N01	A7@N3	0.24
:52@N01	G53@O5′	0.06
:52@N01	G53@OP2	0.03
:52@N01	G10@O4′	0.03
:52@N01	G9@O2′	0.03
:52@N01	A7@N7	0.03
:52@N01	G10@N7	0.01
:52@N01	G10@N9	0.01
:52@N01	U8@O2	0.01

References

(1) Case, D. A.; Cerutti, D. S.; Cruzeiro, V. W. D.; Darden, T. A.; Duke, R. E.; Ghazimirsaeed, M.; Giambaşu, G. M.; Giese, T. J.; Götz, A. W.; Harris, J. A. et al. Recent Developments in Amber Biomolecular Simulations. *J. Chem. Inf. Model.* **2025**, *65*, 7835–7843.

(2) Wang, J.; Cieplak, P.; Kollman, P. A. How well does a restrained electrostatic potential (RESP) model perform in calculating conformational energies of organic biological molecules. *J. Comput. Chem.* **2000**, *21*, 1049–1074.

(3) Case, D. A.; Betz, R. M.; Cerutti, D. S.; Cheatham III, T. E.; Darden, T. A.; Duke, R. E.; Giese, T. J.; Gohlke, H.; Goetz, A. W.; Homeyer, N. et al. AMBER 16. University of California, San Francisco: San Francisco, CA, 2016.

(4) Møller, C.; Plesset, M. S. Note on an approximation treatment for many-electron systems. *Phys. Rev.* **1934**, *46*, 618–622.

(5) Loncharich, R. J.; Brooks, B. R.; Pastor, R. W. Langevin dynamics of peptides: the frictional dependence of isomerization rates of N-acetylalanyl-N'-methylamide. *Biopolymers* **1992**, *32*, 523–535.

(6) Åqvist, J.; Wennerström, P.; Nervall, M.; Bjelic, S.; Brandsdal, B. O. Molecular dynamics simulations of water and biomolecules with a Monte Carlo constant pressure algorithm. *Chem. Phys. Lett.* **2004**, *384*, 288–294.

(7) Lee, T.-S.; Lin, Z.; Allen, B. K.; Lin, C.; Radak, B. K.; Tao, Y.; Tsai, H.-C.; Sherman, W.; York, D. M. Improved Alchemical Free Energy Calculations with Optimized Smoothstep Softcore Potentials. *J. Chem. Theory Comput.* **2020**, *16*, 5512–5525.

(8) Shirts, M. R.; Chodera, J. D. Statistically optimal analysis of samples from multiple equilibrium states. *J. Chem. Phys.* **2008**, *129*, 124105.

(9) Giese, T. J.; York, D. M. FE-ToolKit: The free energy analysis toolkit. https://gitlab.com/RutgersLBSR/fe-toolkit.

(10) Giese, T. J.; Snyder, R.; Piskulich, Z.; Barletta, G. P.; Zhang, S.; McCarthy, E.; Ekesan, Ş.; York, D. M. FE-ToolKit: A Versatile Software Suite for Analysis of High-Dimensional Free Energy Surfaces and Alchemical Free Energy Networks. *J. Chem. Inf. Model.* **2025**, *65*, 5273–5279.

(11) Giese, T. J.; Zeng, J.; York, D. M. Multireference Generalization of the Weighted Thermodynamic Perturbation Method. *J. Phys. Chem. A* **2022**, *126*, 8519–8533.

(12) Mlotkowski, A. J.; Schlegel, H. B.; Chow, C. S. Calculated pK_a Values for a Series of Aza- and Deaza-Modified Nucleobases. *J. Phys. Chem. A* **2023**, *127*, 3526–3534.

(13) Kapinos, L. E.; Operschall, B. P.; Larsen, E.; Sigel, H. Understanding the acid-base properties of adenosine: the intrinsic basicities of N1, N3 and N7. *Chem. Eur. J.* **2011**, *17*, 8156–8164.

(14) Zhao, Y.; Truhlar, D. G. The M06 suite of density functionals for main group thermochemistry, thermochemical kinetics, noncovalent interactions, excited states, and transition elements: two new functionals and systematic testing of four M06-class functionals and 12 other functionals. *Theor. Chem. Acc.* **2008**, *120*, 215–241.

(15) Kendall, R. A.; Dunning, Jr., T. H.; Harrison, R. J. Electron affinities of the first-row atoms revisited. Systematic basis sets and wave functions. *J. Chem. Phys.* **1992**, *96*, 6796–6806.

(16) Adamo, C.; Cossi, M.; Barone, V. An accurate density functional method for the study of magnetic properties: the PBE0 model. *J. Mol. Struct. (Theochem)* **1999**, *193*, 145–157.

(17) Hariharan, P. C.; Pople, J. A. The Influence of Polarization Functions on Molecular Orbital Hydrogenation Energies. *Theor. Chim. Acta* **1973**, *28*, 213–222.

(18) Marenich, A. V.; Cramer, C. J.; Truhlar, D. G. Universal Solvation Model Based on Solute Electron Density and on a Continuum Model of the Solvent Defined by the Bulk Dielectric Constant and Atomic Surface Tensions. *J. Phys. Chem. B* **2009**, *113*, 6378–6396.

(19) Huang, L.; Lilley, D. M. Structure and ligand binding of the SAM-V riboswitch. *Nucleic Acids Res.* **2018**, *46*, 6869–6879.

(20) Huang, L.; Wang, J.; Lilley, D. A critical base pair in k-turns determines the conformational class adopted, and correlates with biological function. *Nucleic Acids Res.* **2016**, *44*, 5390–5398.

(21) Lu, C.; Smith, A. M.; Fuchs, R. T.; Ding, F.; Rajashankar, K.; Henkin, T. M.; Ke, A. Crystal structures of the SAM-III/SMK riboswitch reveal the SAM-dependent translation inhibition mechanism. *Nat. Struct. Mol. Biol.* **2008**, *15*, 1076–1083.

(22) Jiang, H.; Gao, Y.; Zhang, L.; Chen, D.; Gan, J.; Murchie, A. I. H. The identification and characterization of a selected SAM-dependent methyltransferase ribozyme that is present in natural sequences. *Nat. Catal.* **2021**, *4*, 872–881.
